# Zaxinone synthase controls arbuscular mycorrhizal colonization level in rice

**DOI:** 10.1111/tpj.15917

**Published:** 2022-08-17

**Authors:** Cristina Votta, Valentina Fiorilli, Imran Haider, Jian You Wang, Raffaella Balestrini, Ivan Petřík, Danuše Tarkowská, Ondřej Novák, Akmaral Serikbayeva, Paola Bonfante, Salim Al‐Babili, Luisa Lanfranco

**Affiliations:** ^1^ Department of Life Sciences and Systems Biology University of Turin Turin 10125 Italy; ^2^ The BioActives Lab, Center for Desert Agriculture (CDA), Biological and Environment Science and Engineering (BESE) King Abdullah University of Science and Technology Thuwal 23955 Saudi Arabia; ^3^ National Research Council Institute for Sustainable Plant Protection Turin 10135 Italy; ^4^ Laboratory of Growth Regulators, Faculty of Science Palacký University and Institute of Experimental Botany, The Czech Academy of Sciences Olomouc 78371 Czech Republic

**Keywords:** apocarotenoids, arbuscular mycorrhizal symbiosis, GR24, *in situ* hybridization, *OsPT11*, *Oryza sativa*, strigolactones, zaxinone, zaxinone synthase

## Abstract

The *Oryza sativa* (rice) carotenoid cleavage dioxygenase *OsZAS* was described to produce zaxinone, a plant growth‐promoting apocarotenoid. A *zas* mutant line showed reduced arbuscular mycorrhizal (AM) colonization, but the mechanisms underlying this behavior are unknown. Here, we investigated how *OsZAS* and exogenous zaxinone treatment regulate mycorrhization. Micromolar exogenous supply of zaxinone rescued root growth but not the mycorrhizal defects of the *zas* mutant, and even reduced mycorrhization in wild‐type and *zas* genotypes. The *zas* line did not display the increase in the level of strigolactones (SLs) that was observed in wild‐type plants at 7 days post‐inoculation with AM fungus. Moreover, exogenous treatment with the synthetic SL analog GR24 rescued the *zas* mutant mycorrhizal phenotype, indicating that the lower AM colonization rate of *zas* is caused by a deficiency in SLs at the early stages of the interaction, and indicating that during this phase OsZAS activity is required to induce SL production, possibly mediated by the Dwarf14‐Like (D14L) signaling pathway. *OsZAS* is expressed in arbuscule‐containing cells, and *OsPT11*prom::*OsZAS* transgenic lines, where *OsZAS* expression is driven by the *OsPT11* promoter active in arbusculated cells, exhibit increased mycorrhization compared with the wild type. Overall, our results show that the genetic manipulation of OsZAS activity *in planta* leads to a different effect on AM symbiosis from that of exogenous zaxinone treatment, and demonstrate that OsZAS influences the extent of AM colonization, acting as a component of a regulatory network that involves SLs.

## Significance Statement

OsZAS was described to produce zaxinone, a plant growth‐promoting apocarotenoid in *Oryza sativa* (rice). Here we demonstrate the importance of *OsZAS* to guarantee the correct extent of root colonization by arbuscular mycorrhizal (AM) fungi. We show that OsZAS controls the extent of AM colonization, acting as a component of a regulatory network, possibly mediated by the Dwarf14‐Like (D14L) signaling pathway, that involves SLs.

## INTRODUCTION

Most terrestrial plants, including major crops, establish a root mutualistic association called arbuscular mycorrhizal (AM) symbiosis (Genre *et al.*, 2020) with soil fungi belonging to Glomeromycotina (Spatafora *et al.*, 2016). This evolutionarily ancient interaction implies a reciprocal delivery of nutrients: host plants receive mineral nutrients, mainly phosphorus (P), whereas AM fungi rely on plant‐derived fixed carbon (Rich *et al.*, 2017). Additional benefits at organism and ecosystem levels make AM symbiosis a promising component of sustainable agricultural production (Chen *et al.*, 2018; Rillig *et al.*, 2019).

The establishment of AM symbiosis follows a finely tuned colonization pattern. The pre‐symbiotic phase is characterized by a molecular dialog involving the release of diffusible signals (Lanfranco, Fiorilli, & Gutjahr, 2018; Lanfranco, Fiorilli, Venice, & Bonfante, 2018) that leads to the activation of the so‐called common symbiosis signaling pathway (MacLean *et al.*, 2017). Upon reaching the roots epidermis, the fungus develops adhesion structures called hyphopodia that enable the fungus to penetrate host tissues and proliferate *via* intercellular and/or intracellular routes. The symbiotic phase culminates when the fungal hyphae penetrate single cells of the inner cortical layer and form highly branched, tree‐shaped structures, called arbuscules. Arbuscules are always enveloped by a plant‐derived periarbuscular membrane (PAM) that forms an extensive interface for nutrient exchange (Gutjahr & Parniske, 2013). The PAM is indeed populated by a unique set of proteins, such as Pht1 phosphate (Pi) transporters that are responsible for the uptake of Pi delivered by the fungus (Harrison *et al.*, 2002; Yang *et al.*, 2012).

Phytohormones and other signaling molecules have been shown to play a role mainly in the control of the extent of fungal colonization of the root system (Müller & Harrison, 2019). Strigolactones (SLs), a group of carotenoid‐derived hormones, are the best‐known molecules active in early plant–AM fungal interaction (Lanfranco, Fiorilli, & Gutjahr, 2018; Lanfranco, Fiorilli, Venice, & Bonfante, 2018). SLs are produced by roots of Pi‐starved plants and exported to the rhizosphere, where they stimulate AM fungal metabolism, gene expression and hyphal branching, enhancing the chances of the fungus intercepting host plants (Akiyama *et al.*, 2005; Besserer *et al.*, 2006, 2008). However, the dynamics of SL production and their role during the later steps of AM colonization remain elusive.

The involvement of carotenoid metabolism in AM symbiosis is not restricted to SLs and to the early steps of colonization. Indeed, several lines of evidence suggest the initiation and the development of AM symbiosis are influenced by other apocarotenoids (Fiorilli *et al.*, 2019 and reference therein). Among them, the well‐characterized plant hormone abscisic acid (ABA; C_15_) plays key roles in plant response to abiotic stress (Felemban *et al.*, 2019; Peleg & Blumwald, 2011), regulates plant growth and development, and promotes pathogen defense responses (Ma *et al.*, 2018; Ton *et al.*, 2009) and mycorrhizal colonization (Charpentier *et al.*, 2014; Herrera‐Medina *et al.*, 2007; Martín‐Rodríguez *et al.*, 2011). The role of ABA in AM symbiosis remains enigmatic: *Solanum lycopersicum* (tomato) ABA mutants showed reduced levels of AM colonization compared with the wild type; however, in *Medicago truncatula*, ABA treatment promotes AM colonization at low concentrations (Charpentier *et al.*, 2014; Herrera‐Medina *et al.*, 2007; Martín‐Rodríguez *et al.*, 2011). Other works have highlighted an antagonistic interaction between ABA and other hormones involved in AM symbiosis, such as ethylene (Martín‐Rodríguez *et al.*, 2011) and gibberellins (GAs) (Floss *et al.*, 2013; Martín‐Rodríguez *et al.*, 2016).

In addition, other specific classes of apocarotenoids, such as mycorradicins (C_14_) and blumenols (C_13_), are nowadays considered a signature for the establishment of AM symbiosis, as they are specifically accumulated in mycorrhizal plants (Hill *et al.*, 2018; Moreno *et al.*, 2021; Walter *et al.*, 2007; Wang *et al.*, 2018).

The formation of most of the plant apocarotenoid hormones and signaling molecules involves carotenoid cleavage dioxygenases (CCDs), an evolutionarily conserved family of non‐heme Fe^2+^‐dependent enzymes (Giuliano *et al.*, 2003; Hou *et al.*, 2016; Moise *et al.*, 2005; Wang *et al.*, 2021). The recent characterization of a member of the overlooked sixth CCD subfamily led to the identification of zaxinone (3‐OH‐all‐*trans*‐apo‐13‐carotenone), an important growth‐regulating apocarotenoid metabolite in plants (Ablazov *et al.*, 2020; Wang *et al.*, 2019). The enzyme responsible for its biosynthesis in *Oryza sativa* (rice), zaxinone synthase (ZAS), has a wide distribution in the plant kingdom although a homolog gene is absent in the genomes of non‐AM host species, such as *Arabidopsis thaliana* (Wang *et al.*, 2019). A rice mutant (*zas*), defective in *OsZAS*, showed lower zaxinone content and higher levels of SLs in roots, as well as severely retarded root and shoot growth. Exogenous application of zaxinone not only rescued the *zas* root phenotype but also promoted root growth in wild‐type plants and reduced SL biosynthesis and exudation under Pi‐limited and non‐mycorrhizal conditions (Wang *et al.*, 2019). Despite the increased SL content the rice *zas* mutant displayed a reduced, by half, level of AM colonization, compared with wild‐type plants. However, the mechanisms leading to the impaired mycorrhization of the mutant line are not known.

The aim of this study was to understand the role of *OsZAS* and its product zaxinone in the regulation of AM symbiosis. It has been shown that zaxinone has no effect on *Gigaspora margarita* spore germination (Wang *et al.*, 2020), suggesting that perturbation of the fungal asymbiotic phase is unlikely. We therefore hypothesized that zaxinone controls the rate of colonization success through interactions with SLs and other hormones. To address these issues we investigated the phytohormone contents of wild‐type and *zas* genotypes; we performed different exogenous treatments with the aim to restore the expected colonization level in the *zas* mutant. In addition, we analyzed *OsZAS* gene expression at cellular resolution and we characterized transgenic lines in which the expression of *OsZAS* is driven by a promoter active in arbusculated cells (*OsPT11*prom::*OsZAS* lines). Our findings highlight that the SL profiles of wild‐type and *zas* genotypes depend on the plant developmental stage as well as the AM colonization process. In this context we demonstrate that *OsZAS* plays a regulatory role in SL production, possibly through D14‐Like signaling, during the early colonization process and, when expressed under the *OsPT11* promoter, promotes fungal intraradical development.

## RESULTS AND DISCUSSION

### The low colonization level of the *zas* mutant is rescued by SLs, but not by exogenous treatments with zaxinone

**Figure 1:**
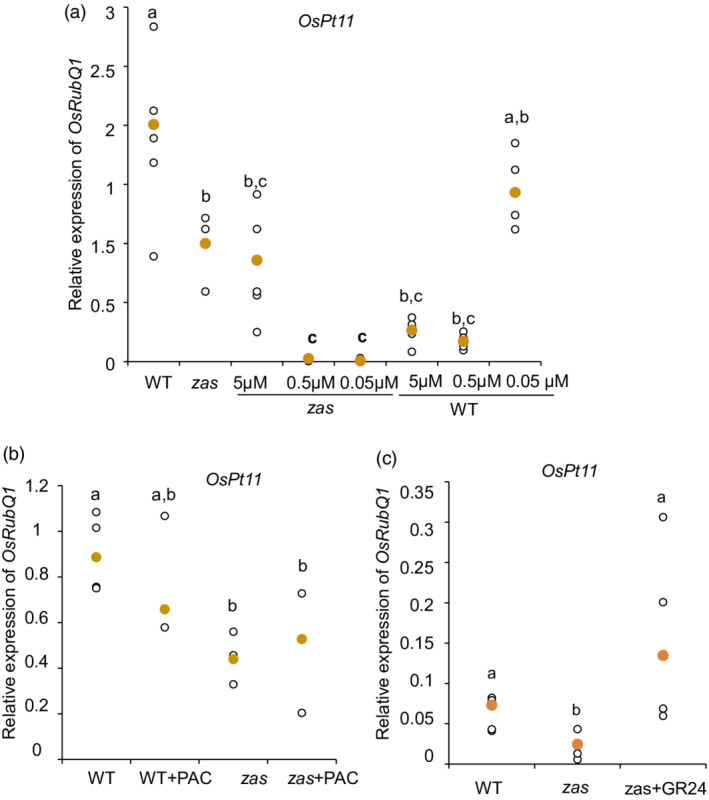
Mycorrhization level in wild‐type and *zas* mutant *Oryza sativa* (rice) plants grown under different treatments, evaluated on the abundance of *phosphate transporter 11* gene (*OsPT11*) transcripts. (a) The relative expression levels of *OsPT11* in mycorrhizal Nipponbare wild‐type (WT) and *zas* mutant (*zas*) roots, treated or not with different zaxinone concentrations (5, 0.5 and 0.05 μm). Mock treatment has the respective % of acetone used for 5‐μm treatments. (b) The relative expression levels of *OsPT11* in mycorrhizal Nipponbare wild‐type (WT) and *zas* mutant (*zas*) roots, treated or not with paclobutrazol (PAC). (c) The relative expression level of *OsPT11* in mycorrhizal roots of wild‐type (WT), *zas* and *zas +* GR24 plants. All plants were harvested at 35 days post inoculation (35 dpi) with *Funneliformis mosseae*. Zaxinone and PAC treatments were performed once a week directly in the nutrient solution, starting 10 days after mycorrhizal inoculation. GR24 (10 nm) treatment was applied once a week directly in the nutrient solution for the entire growing period. *Ubiquitin* was used as a reference gene. Individual data for each condition are shown as white dots and the median values are shown as yellow dots. For each experiment we considered at least *n* = 4 plants. Different letters represent statistically significant differences (*P* < 0.05, one‐way ANOVA; nsd, not statistically different). All experiments were repeated twice with equivalent results.

As the *zas* mutant displayed decreased mycorrhizal colonization (Wang *et al.*, 2019), we tested whether this phenotype could be restored by an exogenous treatment with zaxinone. Therefore, we applied zaxinone at different concentrations (5, 0.5 and 0.05 μm) on 10‐day‐old wild‐type and *zas* mycorrhizal plants. Although the application of zaxinone successfully rescued most plant phenotypic defects in the mutant, i.e. crown root number, shoot length and biomass (Figure S1), it did not restore the expected AM colonization level, as shown by the quantitative reverse transcription polymerase chain reaction (qRT‐PCR) on plant AM marker genes *OsPT11* (Figure 1a) and *OsLysM*, and the fungal *18S* rRNA, and also by a morphological assessment (Figures S2, S3 and S4). In wild‐type plants the lowest zaxinone concentration (0.05 μm) had no impact on mycorrhization but the 0.5 or 5 μm concentrations strongly reduced the AM colonization (Figures 1a, S2, S3 and S4). We hypothesize that the reduced mycorrhization of wild‐type plants might be caused by the negative impact of exogenous zaxinone on SL biosynthesis (Wang *et al.*, 2019) or to alterations to other plant hormones involved in AM symbiosis. As the terpenoid‐derived phytohormones ABA and GAs were shown to play a role in regulating the extent of the AM colonization (Liao *et al.*, 2018; Pozo *et al.*, 2015), we determined their profile in wild‐type and *zas* genotypes in non‐mycorrhizal and mycorrhizal conditions. In non‐mycorrhizal conditions, the *zas* mutant displayed a decrease in ABA level at 10 days post germination (10 dpg) and at 45 dpg, whereas we observed an increase in gibberellin (GA_3_, GA_20_, GA_13_ and GA_29_) content in at least one of the considered time points (Tables S1 and S2). In mycorrhizal plants, *zas* showed an increase in ABA and GA (particularly GA_1_ and GA_20_) content compared with the wild type (Tables S1 and S2). As it has been shown that biologically active GAs suppress arbuscule development and negatively affect the frequency of mycorrhization (Floss *et al.*, 2013), we tested whether an increased level of GA could be responsible for the low level of mycorrhizal colonization in the *zas* mutant. We treated wild‐type and *zas* mycorrhizal plants with paclobutrazol (PAC), which reduces GA levels by inhibiting the ent‐kaurene oxidase/CYP701 (Rademacher, 2000). The effect of the PAC treatment was verified by the plant growth inhibition in both genotypes (Figure S5). In the *zas* mutants, PAC supply rescued neither the growth phenotype nor the mycorrhizal phenotype (Figures 1b, S5 and S6), indicating that the low level of mycorrhization of the mutant line was not cause by a perturbation in GA levels.

So far, the only well‐characterized hormones that promote the establishment of AM symbiosis are SLs, which are active at the early stage of AM interaction (Lanfranco, Fiorilli, Venice, & Bonfante, 2018).

We therefore treated the *zas* mutant with a racemic solution of GR24, an SL synthetic analog, and evaluated the AM colonization at 35 dpi. Notably, the GR24 treatment completely rescued the *zas* mutant mycorrhizal defect (Figures 1c and Figure S7), including the number of hyphopodia and arbuscules that were severely reduced in the untreated *zas* mutant compared with the wild type (Figures 1c and S7). These data suggest that the lower AM colonization rate of *zas* was linked to a deficiency in SLs at the early stage of the AM interaction. In addition, and notably, GR24 treatments did not rescue the *zas* root defects in non‐mycorrhizal (Figure S8) and mycorrhizal (Figure S9) conditions, which were by contrast rescued by the exogenous supply of zaxinone (Wang *et al.*, 2019). These results indicate that the mycorrhizal and root defects of the *zas* mutant could be restored by distinct molecules: SLs and zaxinone, respectively, confirming the prominent role of zaxinone in controlling root development (Wang *et al.*, 2019).

**Figure 2:**
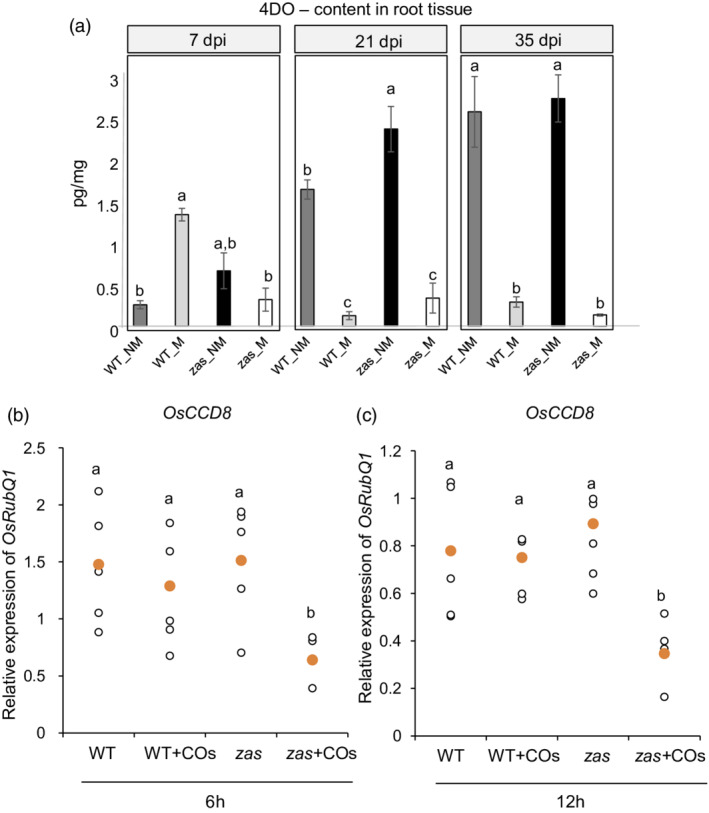
Strigolactone (SL) content and effect of treatment with short‐chain chito‐oligosaccharides (COs) on *OsCCD8* in wild‐type and *zas Oryza sativa* (rice) plants. (a) SL, 4‐deoxyorobanchol (4DO), quantification in wild‐type and *zas* mutant roots in mycorrhizal and non‐mycorrhizal conditions. Roots from mycorrhizal (MYC) and non‐mycorrhizal (NM) plants were collected at 7, 21 and 35 days post fungal inoculation (dpi). Three independent biological replicates (each replicate is a pool of 40 plants) have been considered for the analysis. Different letters represent statistically significant differences within time points (*P* < 0.05, one‐way ANOVA). (b, c) The relative expression level of *OsCCD8* in non‐mycorrhizal roots of wild‐type (WT) and *zas* plants treated (+COs) or not with COs. All plants were harvested at 7 days of growth: (b) after 6 hours post‐COs treatment (hpt); (c) and after 12 hpt. *Ubiquitin* was used as a reference gene (*n* = 5 plants). Different letters represent statistically significant differences (*P* < 0.05, one‐way ANOVA).

We therefore hypothesized a lower SL content in the *zas* mutant during the early stages of AM symbiosis. To investigate this hypothesis we quantified the 4DO content in both genotypes, along a time‐course experiment and during the colonization process. A different trend in 4DO content was observed in non‐mycorrhizal roots, with the 4DO content increasing along with the developmental stages (7, 21 and 35 dpi; Figure 2a). In mycorrhizal roots the highest 4DO content was observed at 7 dpi (Figure 2a), in agreement with the hypothesis that 4DO content facilitates host plant–AM fungus contact during the early stages (López‐Ráez *et al.*, 2015), whereas at later stages (21 and 35 dpi) the 4DO content decreased, as previously observed in different plant species (Figure 2a; Lanfranco, Fiorilli, Venice, & Bonfante, 2018). Concerning the *zas* mutant, in non‐mycorrhizal roots the 4DO content increased over time and a higher 4DO content compared with the wild type was observed at 21 dpi, as described by Wang *et al.* (2019). Notably, this difference was not statistically significant at earlier (7 dpi) and later (35 dpi) developmental stages (Figure 2a). These data indicate that, under our growth conditions, the increase in SL content in the *zas* mutant varies depending on the developmental stage, which is a rather common phenomenon for plant hormones (Rizza & Jones, 2019). In mycorrhizal conditions the 4DO profile of the *zas* mutant was similar to that of wild‐type plants at 21 and 35 dpi, whereas at 7 dpi no 4DO increment was detected (Figure 2a), suggesting that OsZAS activity is involved in the increase in SLs required at this specific stage of the interaction. The different 4DO contents in wild‐type and *zas* mycorrhizal roots at 7 dpi was also supported by the upregulation of *OsCCD8* (*D10*), a key SL biosynthetic gene, which was exclusively observed in wild‐type roots upon AM fungal inoculation (Figure S10). These results suggest the occurrence of a regulatory link between OsZAS function and SL production during the early colonization process. The increase of *OsZAS* mRNA abundance and zaxinone content observed at the early stage of AM colonization in wild‐type mycorrhizal plants (Wang *et al.*, 2019) is also in line with this model.

The increase of SLs induced by the presence of AM fungus seems, therefore, to be dependent on a fully functional *OsZAS*. To follow this hypothesis we investigated the impact of short‐chain chito‐oligosaccharides (COs), the early signaling molecules released by AM fungi that are known to trigger symbiotic responses in the host (Genre *et al.*, 2013; Volpe *et al.*, 2020), on SL biosynthetic gene expression in the wild type and the *zas* mutant. We monitored the expression of *OsCCD8* (Figure 2b,c) and *OsMAX1‐1400* at 6 h (Figure S10b) and 12 h after treatment with COs (hpt) (Figure S10). As previously reported in other host plants (Giovannetti *et al.*, 2015), no differences were observed in wild‐type plants treated with COs, whereas, interestingly, both SL biosynthetic genes were downregulated in the *zas* mutant after the treatment with COs. This accords with a reduced accumulation of SLs in the *zas* mutant at 7 dpi compared with the wild type in mycorrhizal conditions (Figure 2a). Altogether, these data indicate that at the early stage of the AM interaction, *OsZAS* regulates SLs biosynthesis during the plant–fungus molecular dialog.

**Figure 3:**
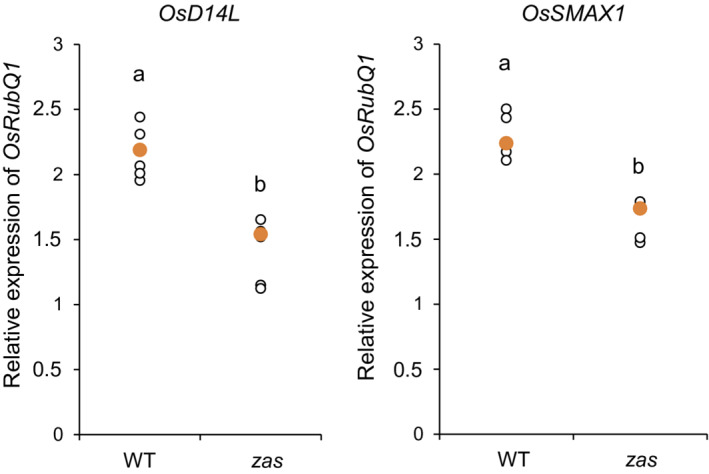
Expression levels of the *Dwarf14‐Like* (D14L) signaling pathway in wild‐type and *zas* mutant *Oryza sativa* (rice) plants. The relative expression levels of (a) *D14L* and (b) *OsSMAX1* in non‐mycorrhizal Nipponbare wild‐type (WT) and *zas* mutant (*zas*) roots at 7 days post growth (dpg). *Ubiquitin* was used as a reference gene (*n* = 5 plants). Different letters represent statistically significant differences (*P* < 0.05, one‐way ANOVA).

The cooperation between SLs and zaxinone biosynthesis during the mycorrhizal colonization of rice is also revealed by recent findings on the signaling pathway mediated by the α/β‐fold hydrolase Dwarf14‐Like (D14L) (Choi *et al.*, 2020), which has been demonstrated to be indispensable for the establishment of AM symbiosis in rice (Gutjahr *et al.*, 2015). It has been shown that D14L signaling positively regulates SL biosynthesis, and therefore AM symbiosis, by eliminating the negative regulator SMAX1 (Choi *et al.*, 2020). Notably, the removal of SMAX1 leads to the upregulation not only of genes involved in SL biosynthesis (i.e. *D10* and *D17*), but also several genes evolutionarily conserved in AM hosts, including *OsZAS*. Therefore, *OsZAS* transcription appears to depend on the activation of the D14L signaling pathway, which is also required to induce SL biosynthetic genes. To support the connection between OsZAS and D14L and SMAX1, we investigated the gene expression level of *OsD14L* and *OsSMAX1* in wild‐type and *zas* genotypes and observed a downregulation of both genes in the mutant compared with wild‐type plants (Figure 3). Intriguingly, these data suggest that the low colonization level of *zas* could also be related to a downregulation of the D14L signaling pathway, which also negatively impacts SL biosynthesis.

Taken as a whole, our data indicate that OsZAS takes part in the mechanisms underpinning the early symbiotic programs that are instrumental in achieving normal mycorrhization levels, influencing both SLs and D14‐L signaling pathways.

### *OsZAS* mRNA is localized in the arbusculated cells

**Figure 4:**
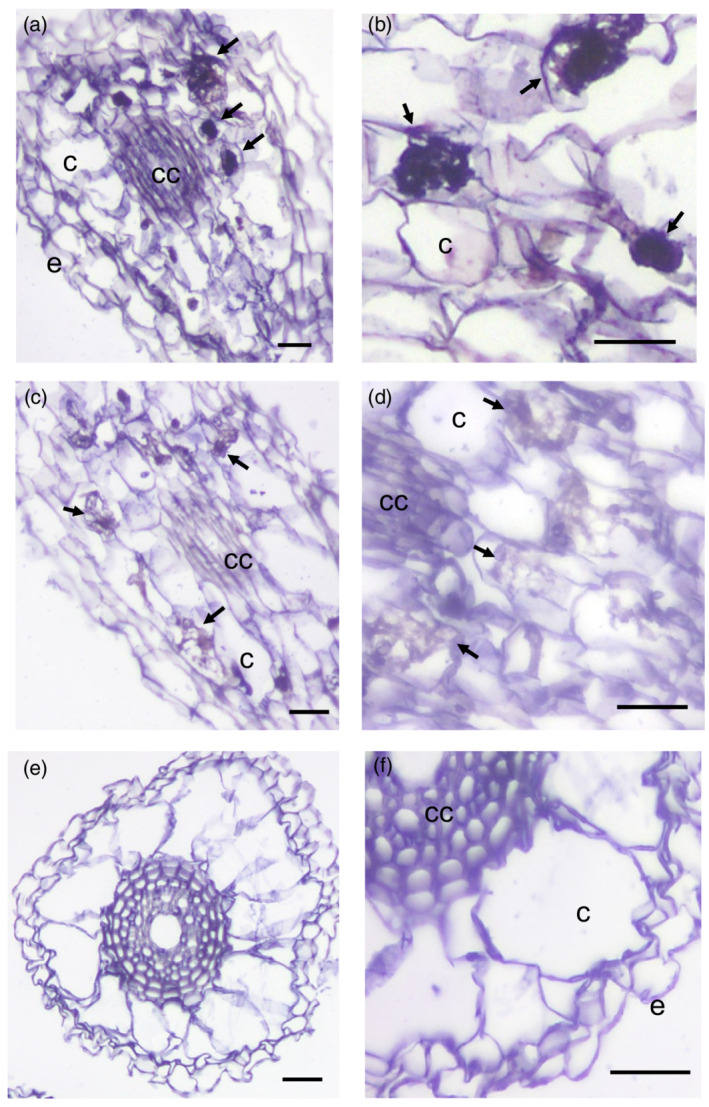
Spatial expression of *OsZAS*. Localization of *OsZAS* mRNA in sections from differentiated regions of inoculated (a–d) and non‐inoculated (e, f) roots of *Oryza sativa* (rice) by cold *in situ* hybridization. (a, b) Section of mycorrhizal roots treated with *OsZAS* antisense probe. A strong chromogenic signal, which mirrors the presence of the *OsZAS* transcripts, is visible in arbuscule‐containing cells (arrows). (c, d) Section of mycorrhizal roots treated with *OsZAS* sense probe, where a hybridization signal is not evident. Arrows indicate the arbuscule‐containing cells that are not labeled. (e, f). Only a very low background is present in uninoculated root segments. c, non‐colonized cortical cells; cc, central cylinder; e, epidermal cells. Scale bars: 50 μm.

We showed that *OsZAS* gene expression is induced in rice roots upon mycorrhizal colonization at 7 and 35 dpi (Wang *et al.*, 2019). With the aim to gain data on *OsZAS* spatial expression, *in situ* hybridization assays were performed on roots of 35‐dpi plants, which correspond to mature mycorrhizas. *OsZAS* mRNA accumulated in cells with fully developed arbuscules where a strong chromogenic signal was observed (Figure 4a,b). By contrast, no signal was detected in non‐colonized cells from mycorrhizal roots (Figure 4a,b) or in sections from mycorrhizal roots hybridized with the *OsZAS* sense probe (Figure 4c,d). Although it was expected to detect *OsZAS* mRNA in non‐mycorrhizal roots, no hybridization signal was observed (Figure 4e,f). We hypothesize that in non‐mycorrhizal cortical cells the level of *OsZAS* mRNA is relatively low, making it undetectable by *in situ* hybridization; an alternative explanation is that *OsZAS* expression is associated with other parts of the root. *OsZAS* spatial expression in mycorrhizal roots is consistent with transcript accumulation observed in the late stages of mycorrhization (Wang *et al.*, 2019) and suggests an involvement of OsZAS in the functioning of arbusculated cells.

### *OsPT11*prom:: *OsZAS* lines show higher root colonization and normal arbuscule morphology

As *in situ* hybridization experiments revealed that *OsZAS* mRNA accumulates in arbusculated cells, we then investigated whether the *OsZAS* expression level has an impact on the intraradical phase and, in particular, on arbuscule formation and development. We thus generated rice transgenic lines, called *OsPT11*prom::*OsZAS* (Figure S11), where *OsZAS* expression is driven by the *OsPT11* promoter that is active in arbuscule‐containing cells (Paszkowski *et al.*, 2002). Two independent, hygromycin‐selected lines, *PT11*prom:*OsZAS_6* (*PT11p::zas6*) and *PT11*prom:*OsZAS_18* (*PT11p::zas18*), were identified by PCR (Figure S11). Both lines were then phenotyped in non‐mycorrhizal (Figure S12) and mycorrhizal conditions (Figure S13). The two lines under non‐mycorrhizal conditions showed an increased crown root length compared with the wild type (Figure S12). This phenotype was similar to that observed in wild‐type plants treated with exogenous zaxinone (Wang *et al.*, 2019). As it has been shown that in the legumes *Medicago truncatula* and *Lotus japonicus* the *OsPT11* homologs are also expressed in root tips when plants are grown under Pi‐limiting conditions (Volpe *et al.*, 2016), we verified whether *OsPT11* was also expressed in rice root apexes. Indeed, *OsPT11* transcripts were detected in root tips of both the wild type and *PT11p::zas6* (Figure S14); notably, we also found that also *OsZAS* is expressed in root apexes and, as expected, transcripts were more abundant in the transgenic line compared with the wild type (Figure S14).

These findings clearly show that in non‐mycorrhizal roots the *OsPT11* promoter is active in root apices, and that the *OsZAS* gene is also expressed in this root tissue. This spatial expression, together with the fact that *OsZAS* expression is induced by Pi starvation (Wang *et al.*, 2019), suggests that OsZAS may be involved in Pi sensing, as has been proposed for the *OsPT11* homologs in legumes (Volpe *et al.*, 2016). The recent discovery that promoters of both *OsZAS* and *OsPT11* genes carry the conserved Pi starvation‐responsive motif P1BS, and are transactivated by the central regulator of Pi signaling, PHR2, has strengthened the idea that these genes have been co‐opted for the Pi sensing pathway and the establishment of AM symbiosis (Das *et al.*, 2021; Shi *et al.*, 2021).

**Figure 5:**
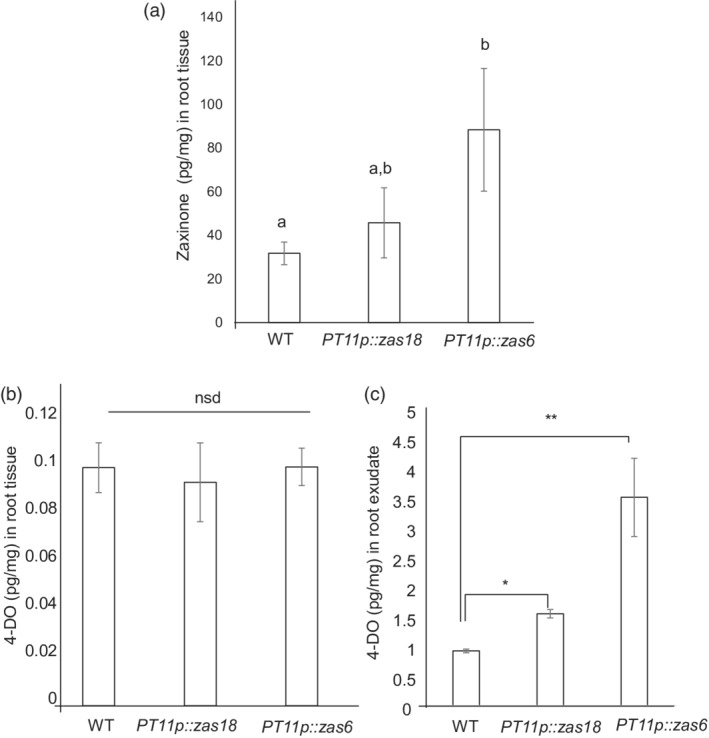
Zaxinone and 4‐deoxyorobanchol (4DO) quantification in *OsPT11*prom*::OsZAS* (*PT11p::ZAS*) lines of *Oryza sativa* (rice). Zaxinone (a) and 4DO content in root tissue (b) and in root exudate (c) were quantified in wild‐type (WT) and *OsPT11*prom*::OsZAS* (*PT11p::ZAS*) lines in non‐mycorrhizal conditions at 21 days post‐germination. Means and standard errors of four biological replicates are shown. Different letters indicate significant differences (*P < 0.05, **P < 0.01).

Moreover, the enhanced growth observed in *OsPT11*prom::*OsZAS* non‐mycorrhizal roots could be the result of localized *OsZAS* upregulation. Indeed, a higher zaxinone content was detected in roots of the non‐mycorrhizal *PT11p::zas6* line, whereas the *PT11p::zas18* line showed a higher but not statistically different level compared with the wild type (Figure 5a). At the same growth stage non‐mycorrhizal wild‐type and *OsPT11*prom::*OsZAS* plants showed similar levels of SLs in the roots (Figure 5b). Remarkably, a higher trend of 4DO level was detected in root exudates of both transgenic lines compared with the wild type *(*Figure 5c).

**Figure 6:**
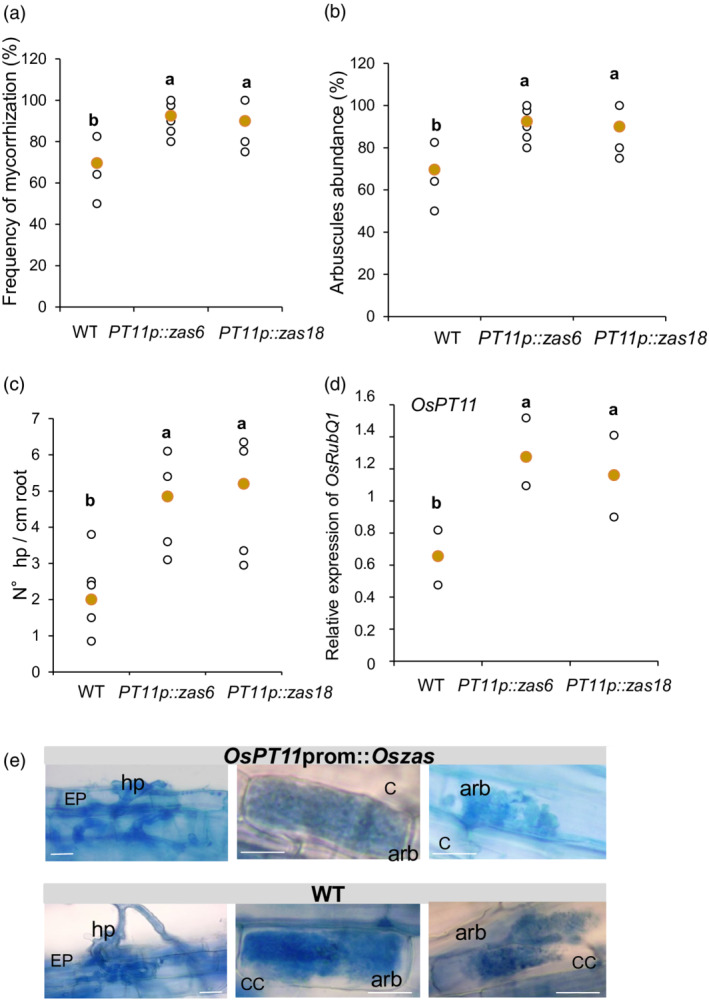
Molecular and phenotypic evaluation of mycorrhizal colonization in *OsPT11*prom*::OsZAS* lines of *Oryza sativa* (rice). After blue cotton staining: (a) the frequency of mycorrhizal (F%) colonization; (b) arbuscule abundance (A%); and (c) number of hyphopodia per cm of roots were evaluated in the wild‐type (WT) and *OsPT11*prom*::OsZAS* lines (*PT11p::zas6*; *PT11p::zas18*) (*n* = 5 plants). (d) The relative expression levels of *OsPT11* in mycorrhizal wild‐type (WT) and *OsPT11*prom*::OsZAS* lines (*PT11p::zas6*; *PT11p::zas18*). *Ubiquitin* was used as a reference gene (*n* = 4 plants). (e) Root epidermal cells (EP) and cortical cells (CC) from wild‐type (WT) and *OsPT11*prom*::OsZAS* lines where hyphopodia (hp) and arbuscules (arb) are shown, respectively; the blue color indicates the cotton blue staining. Scale bars: 80 μm. All plants were harvested 21 days post inoculation with *Funneliformis mosseae*. Individual data for each condition are shown as white dots and median values are shown as yellow dots. Different letters represent statistically significant differences (*P* < 0.05, one‐way ANOVA).

It has been shown that hyphopodium formation is severely attenuated in SL‐deficient *d17* (*CCD7*) *d10* (*CCD8*) rice double mutants, suggesting that a continuous requirement of SLs is essential for hyphopodia formation and the promotion of secondary infection (Kobae *et al.*, 2018). In accordance with these data, *OsPT11*prom::*OsZAS* lines showed an increased AM colonization level in terms of mycorrhization frequency, number of arbuscules and number of hyphopodia, compared with the wild type (Figure 6a–c), whereas the arbuscule morphology was unaltered (Figure 6e). The morphological results were also confirmed by gene expression analyses in the plant *OsPT11* (Figure 6d).

The data obtained from *OsPT11*prom::*OsZAS* lines confirmed the role of *OsZAS* in promoting the AM colonization level, probably by inducing SL biosynthesis, which triggers hyphopodia formation that in turn promotes arbuscule formation; they also provide evidence that localized *OsZAS* overexpression in arbusculated cells does not have an impact on intracellular fungal morphology (Figure 6e).

## CONCLUSION

**Figure 7:**
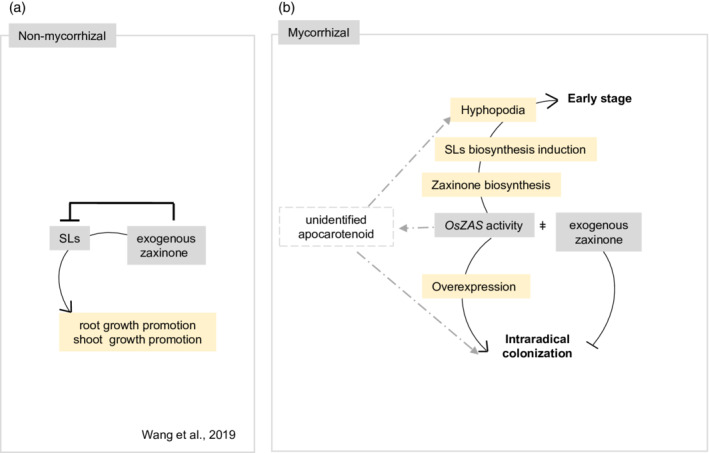
Schematic model for OsZAS and exogenous zaxinone regulation in wild‐type *Oryza sativa* (rice) plants grown in non‐mycorrhizal (a) and mycorrhizal (b) conditions. (a) In low‐Pi conditions, exogenous zaxinone treatment negatively regulates rice SL biosynthesis and release. The zaxinone root and shoot growth promotion requires functional strigolactone (SL) biosynthesis (Wang et al., 2019). (b) OsZAS activity increases zaxinone content and promotes the biosynthesis of SLs and hyphopodia formation in the early stage of mycorrhizal colonization. Overexpression of *OsZAS* under the *OsPT11* promoter increases the intraradical colonization. By contrast, exogenous application of zaxinone negatively impact AM colonization. The discrepancy between the impacts on AM symbiosis of exogenous and endogenous (by OsZAS localized overexpression) accumulation of zaxinone suggests that, besides zaxinone, OsZAS can form *in planta* a yet unidentified apocarotenoid required for optimal mycorrhization (dashed arrow and lines). Positive and negative effects are illustrated by arrows and blunt‐ended bars, respectively.

Overall, the data we present here demonstrate the importance of *OsZAS* to guarantee the correct extent of AM root colonization. In the early stages of the AM interaction, OsZAS modulates AM colonization and exerts its function as a component of a regulatory network that involves SLs and D14L pathways. The overexpression of *OsZAS* in arbusculated cells (*OsPT11*prom::*OsZAS* lines) leads to increased mycorrhization, including an increased abundance of hyphopodia and arbuscules (Figure 7), that could be related to the higher content of SLs in the *OsPT11*prom::*OsZAS* root exudates.

Our results show that the genetic manipulation of OsZAS activity *in planta* leads to a different effect on the AM symbiosis from that of an exogenous zaxinone treatment. Although we found a clear positive correlation between the expression level of *OsZAS* and the extent of colonization, exogenous zaxinone repressed the AM symbiosis, probably through the strong negative impact of a continuous application of this compound on SL biosynthesis (Wang *et al.*, 2019; Figure 7). This highlights that appropriate levels of this apocarotenoid are needed to assist root colonization by AM fungi and that OsZAS activity is involved in a complex network that could not be mimicked by an exogenous supply of its product. One could also speculate that, besides zaxinone, OsZAS can form *in planta* a yet unidentified apocarotenoid required for optimal mycorrhization.

## EXPERIMENTAL PROCEDURES

### Plant and fungal material

For all the experiments, seeds of the Nipponbare wild‐type cultivar, the mutants *zas* (Wang *et al.*, 2019) and *d17* (Butt *et al.*, 2018), and two independent *OsPT11*prom*::Oszas* lines (see Supplemental methods) cv. Nipponbare were germinated as described by Fiorilli *et al.* (2015). Mycorrhizal plants were colonized with *Funneliformis mosseae* (BEG 12; MycAgroLab, http://www.mycagrolab.com) using a fungal inoculum mixed (25%) with sterile quartz sand. Plants were grown and watered as described by Vallino *et al.* (2014). Mycorrhizal roots were stained with cotton blue and the level of mycorrhizal colonization was assessed according to the method described by Trouvelot *et al.* (1986). Hyphopodia were counted manually in each root section.

### *In situ* hybridization and detection

To generate the probe for *in situ* hybridization, a primer pair (Table S3) was used to amplify an *Oszas* sequence of 470 bp. The amplicon was cloned in the sense and antisense orientation into the pCR2.1‐TOPO (TA Cloning^®^; ThermoFisher Scientific, https://www.thermofisher.com) with respect to the T7 promoter. Digoxigenin‐labeled RNA probes were synthesized from PCR fragments with T7 or SP6 RNA polymerase, as described by Balestrini *et al.* (1997). Root segments of 1 cm in length were fixed in 4% paraformaldehyde in PBS overnight at 4°C. Samples were then dehydrated in an ethanol series, embedded in Paraplast Plus and sectioned to 8‐μm thickness using a rotary microtome. *In situ* hybridization and color development were performed as described by Balestrini *et al.* (1997) (see Supplemental methods). Sections were observed under a light microscope (Primo Star Zeiss; Carl Zeiss, https://www.zeiss.com) with a Leica DFC425 digital camera (Leica Microsystems, https://www.leica‐microsystems.com). The experiment was repeated twice with equivalent results.

### Phytohormone quantification

For the quantification of targeted plant hormones and related compounds, 20‐mg (fresh weight) portions of separately harvested roots were frozen in liquid nitrogen. Concentration levels of endogenous phytohormones (ABA and GAs) were determined in four biological replicates according to the modified method described by Šimura *et al.* (2018) (see Supplemental methods).

To measure SLs and zaxinone contents, the protocol described by Wang *et al.* (2019) was followed for different stages of mycorrhizal symbiosis in a time‐course experiment: plants inoculated or not with *F. mosseae* were sampled at 7, 20 and 35 dpi. To measure SL content in root exudate of *OsPT11*prom::*Oszas* lines, the protocol described by Wang *et al.* (2019) was followed.

### Plant treatments

For zaxinone treatment, a set of wild‐type and *zas* mycorrhizal plants were watered twice a week, once by applying 5, 0.5 or 0.05 μm of the compound in the nutrient solution, starting at 10 dpi to avoid a decrease of SL content during the early phase of AM symbiosis. For SL treatment, 10 nm of the SL analog GR24 (racemic solution) was applied once a week on non‐mycorrhizal and mycorrhizal wild‐type and *zas* plants. Both zaxinone and GR24 were dissolved in acetone. For treatment with paclobutrazol (PAC), an inhibitor of GA biosynthesis, 10 μm PAC was applied 10 days after AM fungal inoculation once a week for a total period of 4 weeks.

For the chitooligosaccharides (COs; CO4–CO5) treatment, rice seeds of wild‐type and *zas* mutant plants were germinated in pots containing sand and incubated for 10 days in a growth chamber under a 14‐h light (23°C)/10‐h dark (21°C) photoperiod. Seedlings were transferred to 5‐ml Eppendorf tubes (https://www.eppendorf.com) and were grown hydroponically in a modified Long Ashton (LA) solution containing 3.2 μm Na_2_HPO_4_·12H_2_O. A set of wild‐type (WT + CO) and *zas* (*zas* + CO) plants were treated with a concentration of 10^−5^ M (Carotenuto *et al.*, 2017) of COs mix, previously withthe protocol from Crosino *et al.* (2021), for 6 and 12 h, and then roots were collected for gene expression analysis.

### Nucleic acid extraction and cDNA synthesis

Total RNA was extracted from rice roots using the Plant RNeasy Kit (Qiagen, https://www.qiagen.com). Samples were treated with TURBO™ DNase (Ambion, now ThermoFisher Scientific, https://www.thermofisher.com). The RNA samples were routinely checked for DNA contamination using PCR analysis, using primers for *OsRubQ1* (Güimil *et al.*, 2005). For single‐strand cDNA synthesis, about 1000 ng of total RNA was reverse‐transcribed using Super‐Script II (Invitrogen, now ThermoFisher Scientific, https://www.thermofisher.com).

### Real‐time quantitative RT‐PCR

Quantitative RT‐PCR (qRT‐PCR) was performed using a Rotor‐Gene Q 5plex HRM Platform (Qiagen). Each PCR reaction was carried out as described by Fiorilli *et al.* (2015). All reactions were performed on at least four biological and two technical replicates. The transcript levels of rice *OsPT11* (Güimil *et al.*, 2005), *OsLysM* (Fiorilli *et al.*, 2015), *OsCCD8* and *OsMAX1* (Wang *et al.*, 2019), and *OsD14L* (Gutjahr *et al.*, 2015) and *OsSMAX1* (Choi *et al.*, 2020) and fungal housekeeping *Fm18S* (Balestrini *et al.*, 2007) were normalized using the *OsRubQ1* housekeeping gene (Table S3).

### Statistics

Statistical tests were carried out through one‐way analysis of variance (one‐way ANOVA) and Tukey's *post hoc* test, using a probability level of *P* < 0.05. All statistical elaborations were performed using past 2.16 (Hammer *et al.*, 2001).

## AUTHOR CONTRIBUTIONS

VF, SA‐B, PB and LL designed the investigation. CV and VF performed the cellular and molecular experiments concerning mycorrhization. RB contributed with the *in situ* hybridization. JYW carried out the quantification of zaxinone and SLs. IH and AS generated the transgenic lines. IP, DT and ON conducted the quantification of hormones. All authors contributed to the results and discussion, and VF, SA‐B, PB and LL wrote the article.

## CONFLICT OF INTEREST

The authors declare that they have no conflicts of interest associated with this work.

## Supporting information


**Figure S1.** Phenotypic evaluation of sand‐grown *Oryza sativa* (rice) wild‐type (WT) and *zas* mycorrhizal plants treated with 5 μm zaxinone.
**Figure S2.** Analysis of arbuscular mycorrhizal level in wild‐type (WT) and *zas* mutant plants under 5 μm zaxinone treatment.
**Figure S3.** Analysis of arbuscular mycorrhizal level in wild‐type (WT) and *zas* mutant plants under 0.5 μm zaxinone treatment.
**Figure S4.** Analysis of arbuscular mycorrhizal level in wild‐type (WT) and *zas* mutant plants under 0.05 μm zaxinone treatment.
**Figure S5.** Phenotypic evaluation of sand‐grown *Oryza sativa* (rice) wild‐type (WT) and *zas* plants treated or not with 10 μm paclobutrazol (PAC).
**Figure S6.** Analysis of arbuscular mycorrhizal level in wild‐type (WT) and *zas* mutant plants under 10 μm paclobutrazol (PAC) treatment.
**Figure S7.** Analysis of arbuscular mycorrhizal level in wild‐type (WT) and *zas* mutant plants under 10 nm GR24 treatment.
**Figure S8.** Effect of GR24 treatment on the shoot and root phenotypes of wild‐type (WT) and *zas* mutant plants grown in non‐mycorrhizal conditions.
**Figure S9.** Effect of GR24 treatment on the shoot and root phenotypes of wild‐type (WT) and *zas* mutant plants in mycorrhizal conditions.
**Figure S10.** Relative expression level of SL biosynthesis genes (*OsCCD8* and *OsMAX1*) in wild‐type (WT) and *zas* mutant plants in the early stage of the AM interaction; the number of hyphopodia per cm of root evaluated in WT, *zas* and *zas +* GR24 plants at 35 dpi; and relative expression level of *OsMAX1‐1400* in non‐mycorrhizal roots of wild type (WT) and *zas* mutant plants treated (+COs) or not with COs.
**Figure S11.** Molecular analysis of the *OsPT11prom:OsZAS* transgenic *Oryza sativa* (rice) lines.
**Figure S12.** Phenotypic evaluation of *Oryza sativa* (rice) wild‐type (WT) and *OsPT11*prom*::OsZAS* lines grown in sand in non‐mycorrhizal conditions.
**Figure S13.** Phenotypic evaluation of *Oryza sativa* (rice) wild‐type (WT) and *OsPT11*prom*::OsZAS* lines in mycorrhizal conditions grown in sand at 21 days post‐inoculation.
**Figure S14.** Gel electrophoresis of RT‐PCR products obtained from RNA of root apexes of non‐mycorrhizal wild‐type (WT) and *OsPT11*prom*::OsZAS*(*Pt11_6*) line samples using specific primers.
**Table S1.** ABA quantification (pmol/gFW) in wild‐type (WT) and *zas* non‐mycorrhizal and mycorrhizal roots in a time‐course experiment.
**Table S2.** Gibberellin quantification (pmol/gFW) in wild‐type (WT) and *zas* non‐mycorrhizal and mycorrhizal roots.
**Table S3.** Primer sequences used in this study.

Supplemental methods

## Data Availability

The data that support the findings of this study are available from the corresponding authors, upon reasonable request.
