# Supplementary material for: Zaxinone synthase controls arbuscular mycorrhizal colonization level in rice
Source: Plant J. 2022 Aug 17;111(6):1688–700. doi: 10.1111/tpj.15917 (PMC9543690; doi:10.1111/tpj.15917)
Supplement: Supplementary file 1 — Figure S1. Phenotypic evaluation of sand‐grown Oryza sativa (rice) wild‐type (WT) and zas mycorrhizal plants treated with 5 μm zaxinone. Figure S2. Analysis of arbuscular mycorrhizal level in wild‐type (WT) and zas mutant plants under 5 μm zaxinone treatment. Figure S3. Analysis of arbuscular mycorrhizal level in wild‐type (WT) and zas mutant plants under 0.5 μm zaxinone treatment. Figure S4. Analysis of arbuscular mycorrhizal level in wild‐type (WT) and zas mutant plants under 0.05 μm zaxinone treatment. Figure S5. Phenotypic evaluation of sand‐grown Oryza sativa (rice) wild‐type (WT) and zas plants treated or not with 10 μm paclobutrazol (PAC). Figure S6. Analysis of arbuscular mycorrhizal level in wild‐type (WT) and zas mutant plants under 10 μm paclobutrazol (PAC) treatment. Figure S7. Analysis of arbuscular mycorrhizal level in wild‐type (WT) and zas mutant plants under 10 nm GR24 treatment. Figure S8. Effect of GR24 treatment on the shoot and root phenotypes of wild‐type (WT) and zas mutant plants grown in non‐mycorrhizal conditions. Figure S9. Effect of GR24 treatment on the shoot and root phenotypes of wild‐type (WT) and zas mutant plants in mycorrhizal conditions. Figure S10. Relative expression level of SL biosynthesis genes (OsCCD8 and OsMAX1) in wild‐type (WT) and zas mutant plants in the early stage of the AM interaction; the number of hyphopodia per cm of root evaluated in WT, zas and zas + GR24 plants at 35 dpi; and relative expression level of OsMAX1‐1400 in non‐mycorrhizal roots of wild type (WT) and zas mutant plants treated (+COs) or not with COs. Figure S11. Molecular analysis of the OsPT11prom:OsZAS transgenic Oryza sativa (rice) lines. Figure S12. Phenotypic evaluation of Oryza sativa (rice) wild‐type (WT) and OsPT11prom::OsZAS lines grown in sand in non‐mycorrhizal conditions. Figure S13. Phenotypic evaluation of Oryza sativa (rice) wild‐type (WT) and OsPT11prom::OsZAS lines in mycorrhizal conditions grown in sand at 21 days post‐inoculation. [file TPJ-111-1688-s001.pdf]

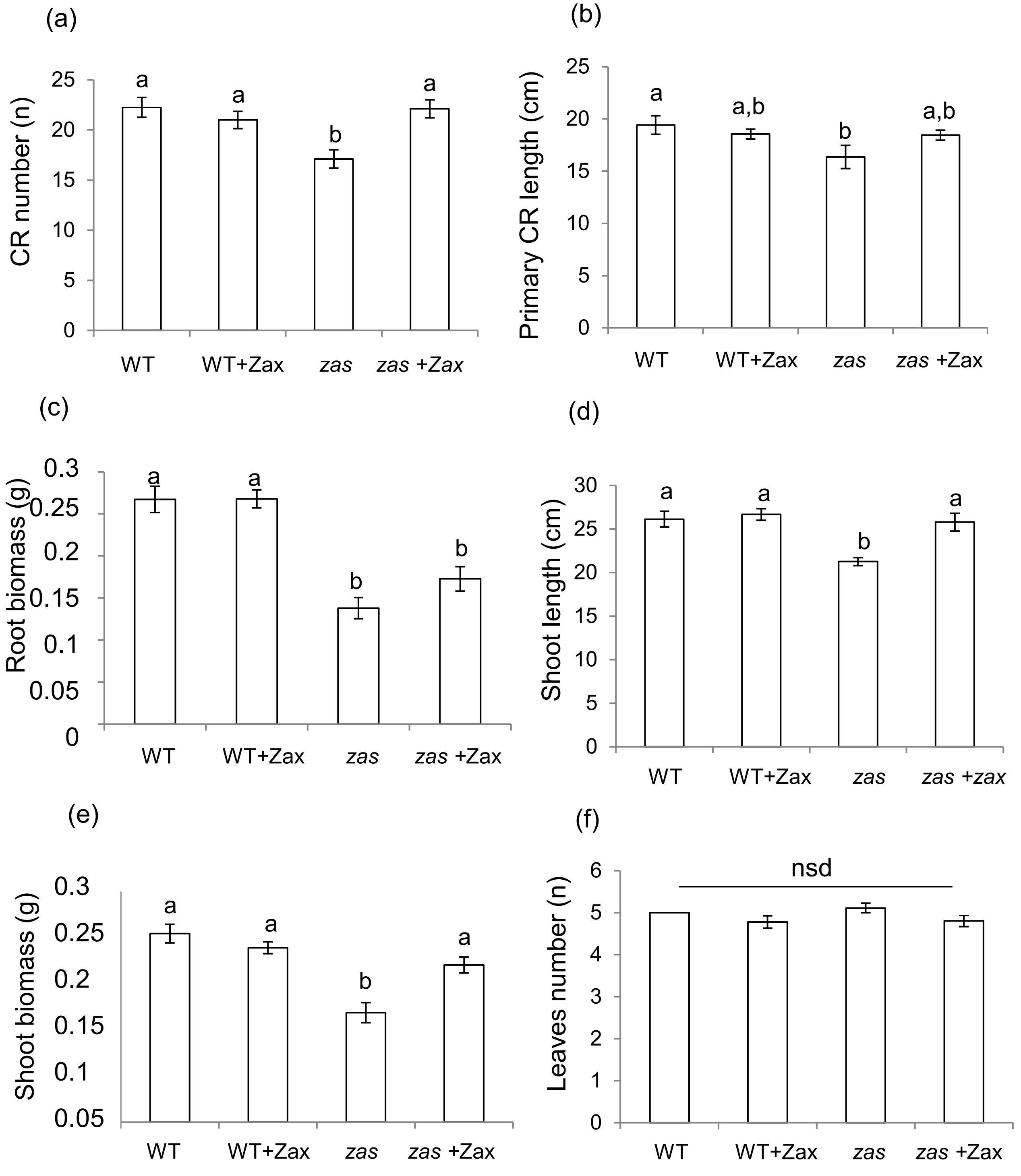
(a)
CR number (n)
WT
WT+Zax
zas
zas +Zax
(b)
Primary CR length (cm)
(c)
Root biomass (g)
(d)
Shoot length (cm)
zas +zax
(e)
Shoot biomass (g)
(f)
Leaves number (n)
nsd

Figure S1

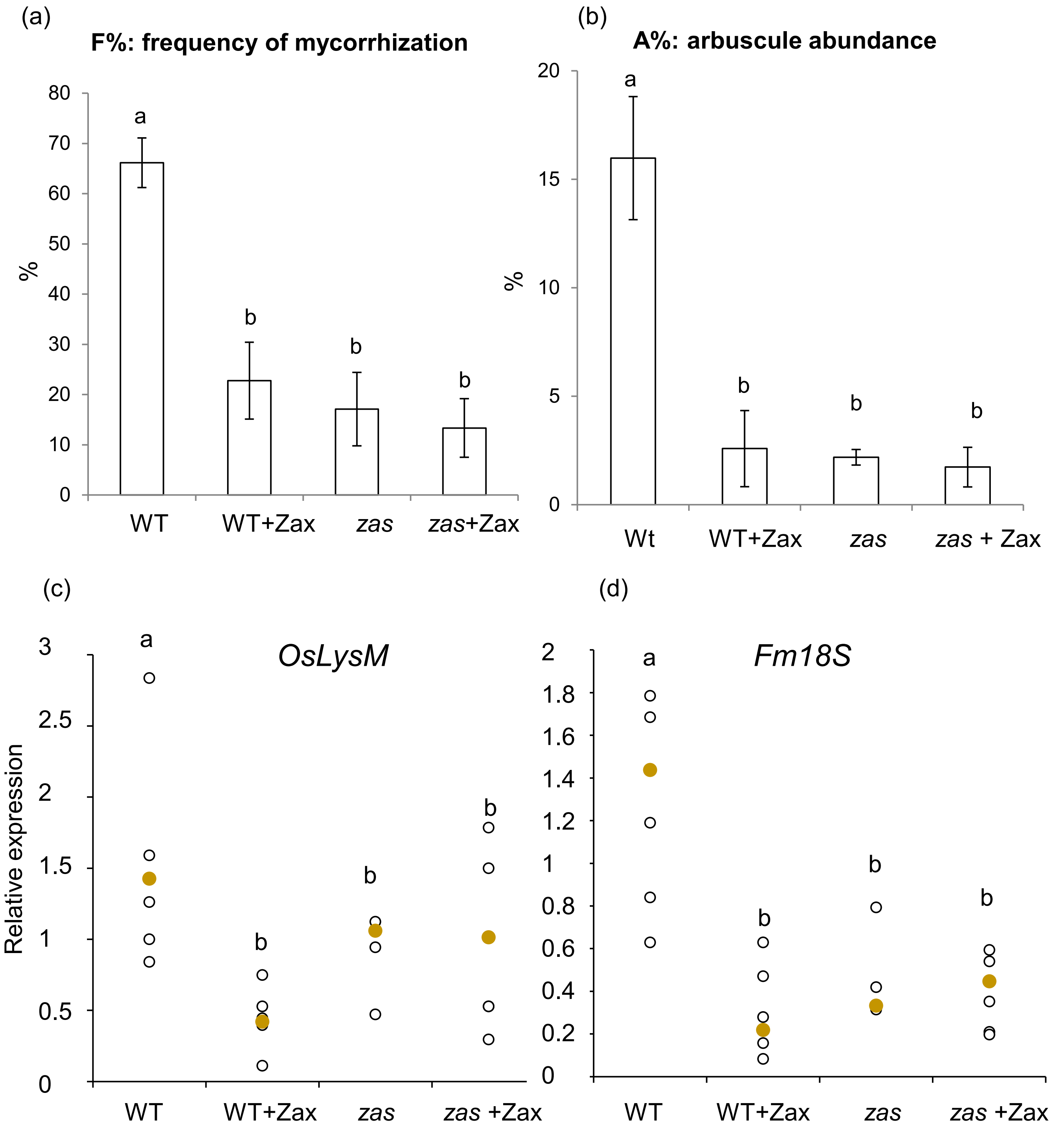

(a)
F%: frequency of mycorrhization
%
80
70
60
50
40
30
20
10
0
a
b
b
b
WT
WT+Zax
zas
zas+Zax
(b)
A%: arbuscule abundance
%
20
15
10
5
0
a
b
b
b
Wt
WT+Zax
zas
zas + Zax
(c)
OsLysM
Relative expression
3
2.5
2
1.5
1
0.5
0
a
b
b
b
WT
WT+Zax
zas
zas +Zax
(d)
Fm18S
2
1.8
1.6
1.4
1.2
1
0.8
0.6
0.4
0.2
0
a
b
b
b
WT
WT+Zax
zas
zas +Zax


Figure S2

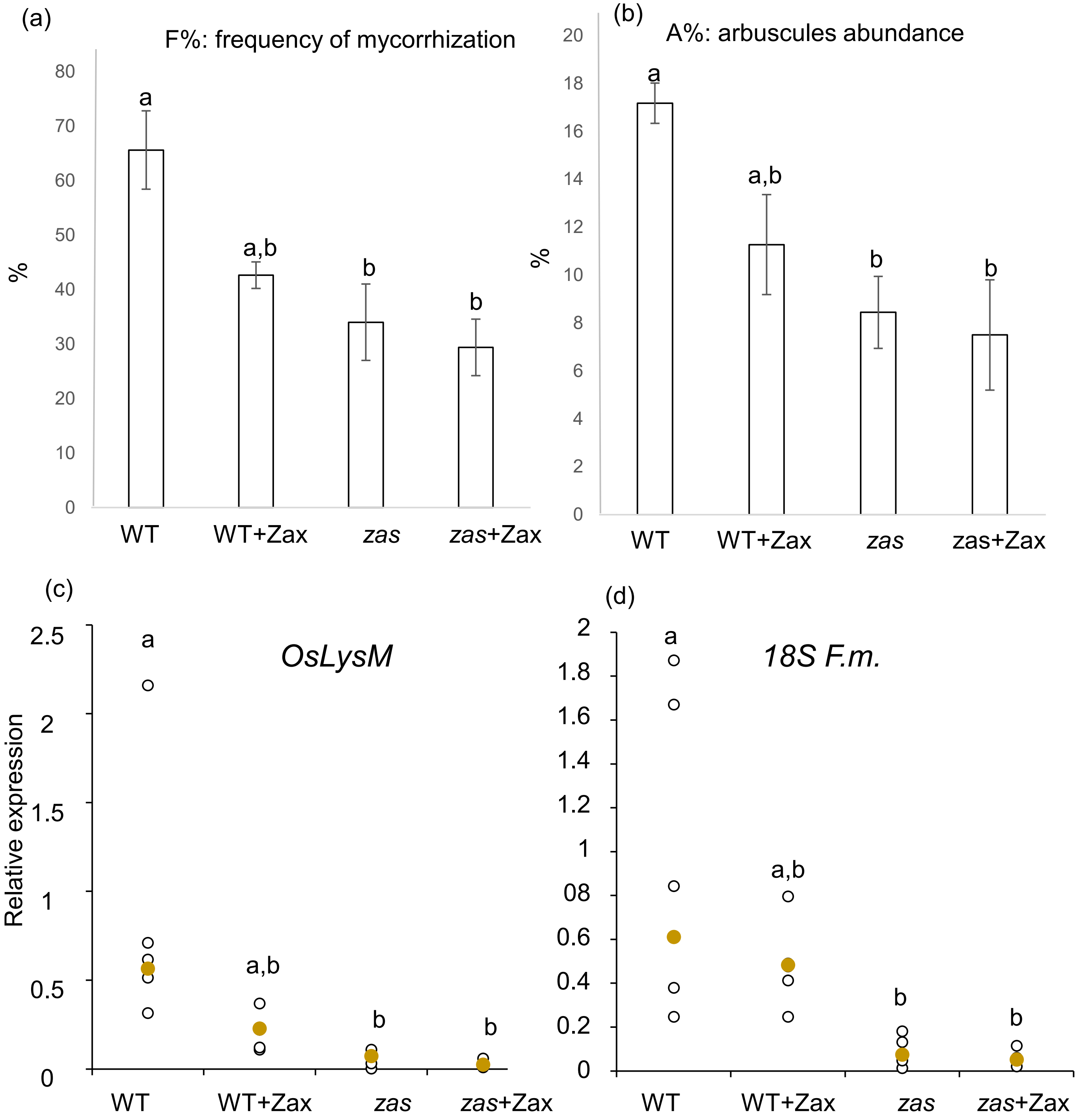
(a)
F%: frequency of mycorrhization
%
80
70
60
50
40
30
20
10
0
a
a,b
b
b
WT
WT+Zax
zas
zas+Zax
(b)
A%: arbuscules abundance
%
20
18
16
14
12
10
8
6
4
2
0
a
a,b
b
b
WT
WT+Zax
zas
zas+Zax
(c)
OsLysM
Relative expression
2.5
2
1.5
1
0.5
0
a
a,b
b
b
WT
WT+Zax
zas
zas+Zax
(d)
18S F.m.
2
1.8
1.6
1.4
1.2
1
08
0.6
0.4
0.2
0
a
a,b
b
b
WT
WT+Zax
zas
zas+Zax

Figure S3

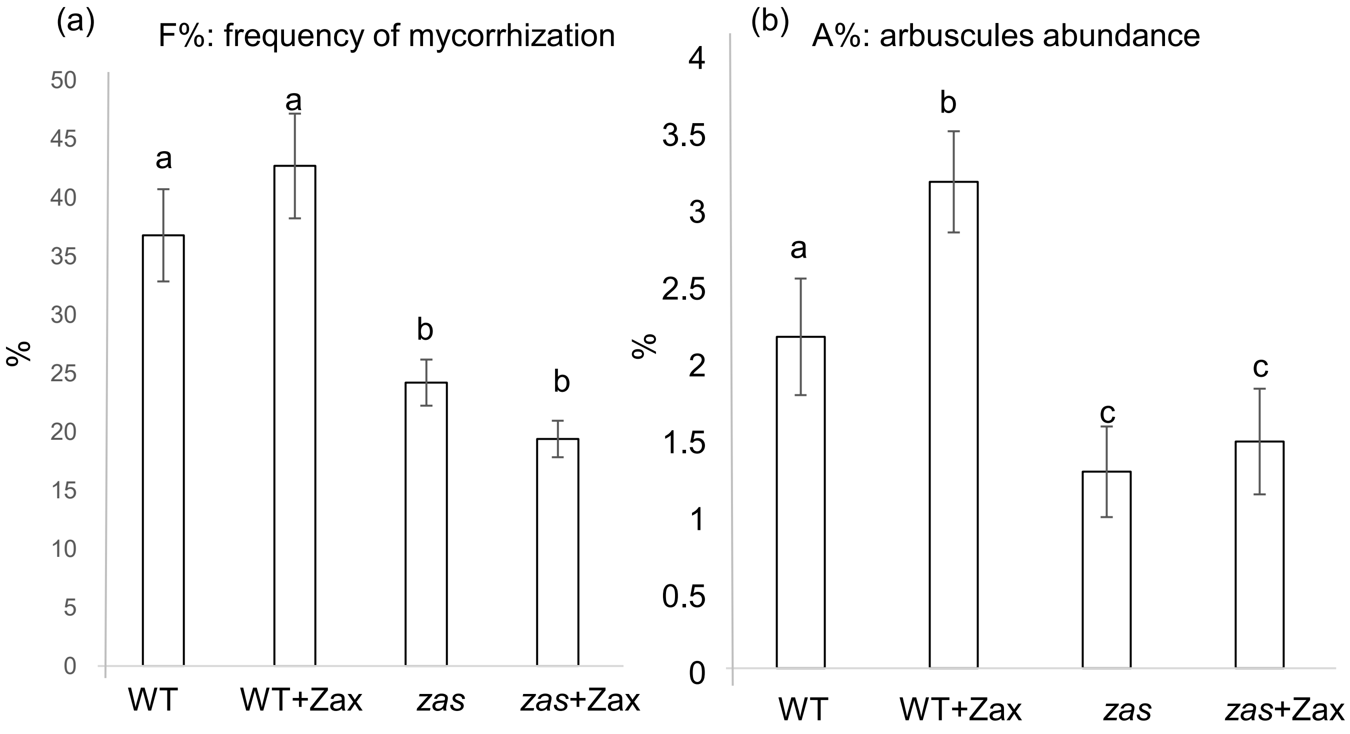

(a)
F%: frequency of mycorrhization
50
45
40
35
30
25
20
15
10
5
0
%
a
a
b
b
WT
WT+Zax
zas
zas+Zax
(b)
A%: arbuscules abundance
4
3.5
3
2.5
2
1.5
1
0.5
0
%
a
b
c
c
WT
WT+Zax
zas
zas+Zax


Figure S4

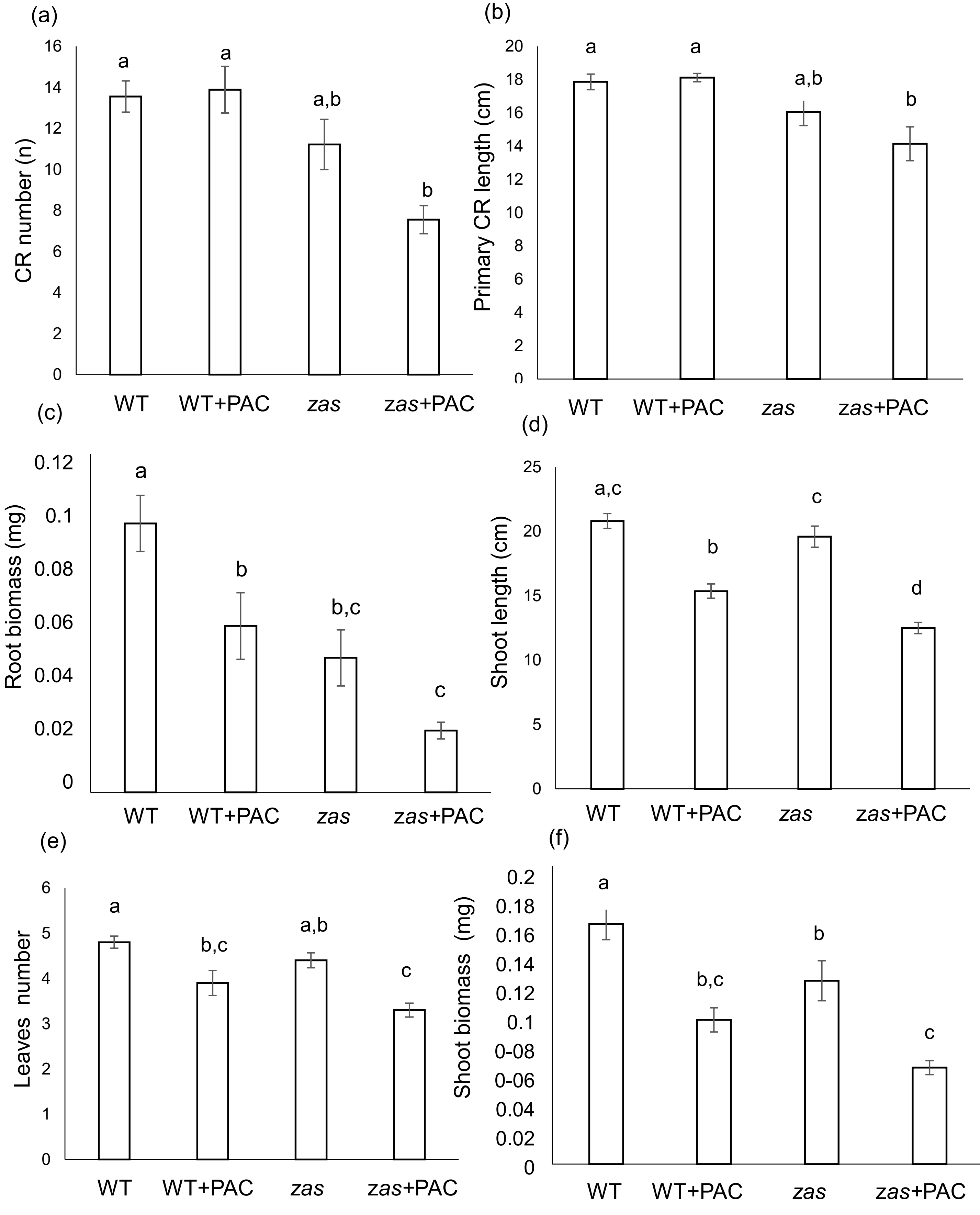
(a)
CR number (n)
16
14
12
10
8
6
4
2
0
a
a
a,b
b
WT
WT+PAC
zas
zas+PAC
(b)
Primary CR length (cm)
20
18
16
14
12
10
8
6
4
2
0
a
a
a,b
b
WT
WT+PAC
zas
zas+PAC
(c)
Root biomass (mg)
0.12
0.1
0.08
0.06
0.04
0.02
0
a
b
b,c
c
WT
WT+PAC
zas
zas+PAC
(d)
Shoot length (cm)
25
20
15
10
5
0
a,c
b
c
d
WT
WT+PAC
zas
zas+PAC
(e)
Leaves number
6
5
4
3
2
1
0
a
b,c
a,b
c
WT
WT+PAC
zas
zas+PAC
(f)
Shoot biomass (mg)
0.2
0.18
0.16
0.14
0.12
0.1
0-08
0-06
0.04
0.02
0
a
b,c
b
c
WT
WT+PAC
zas
zas+PAC

Figure S5

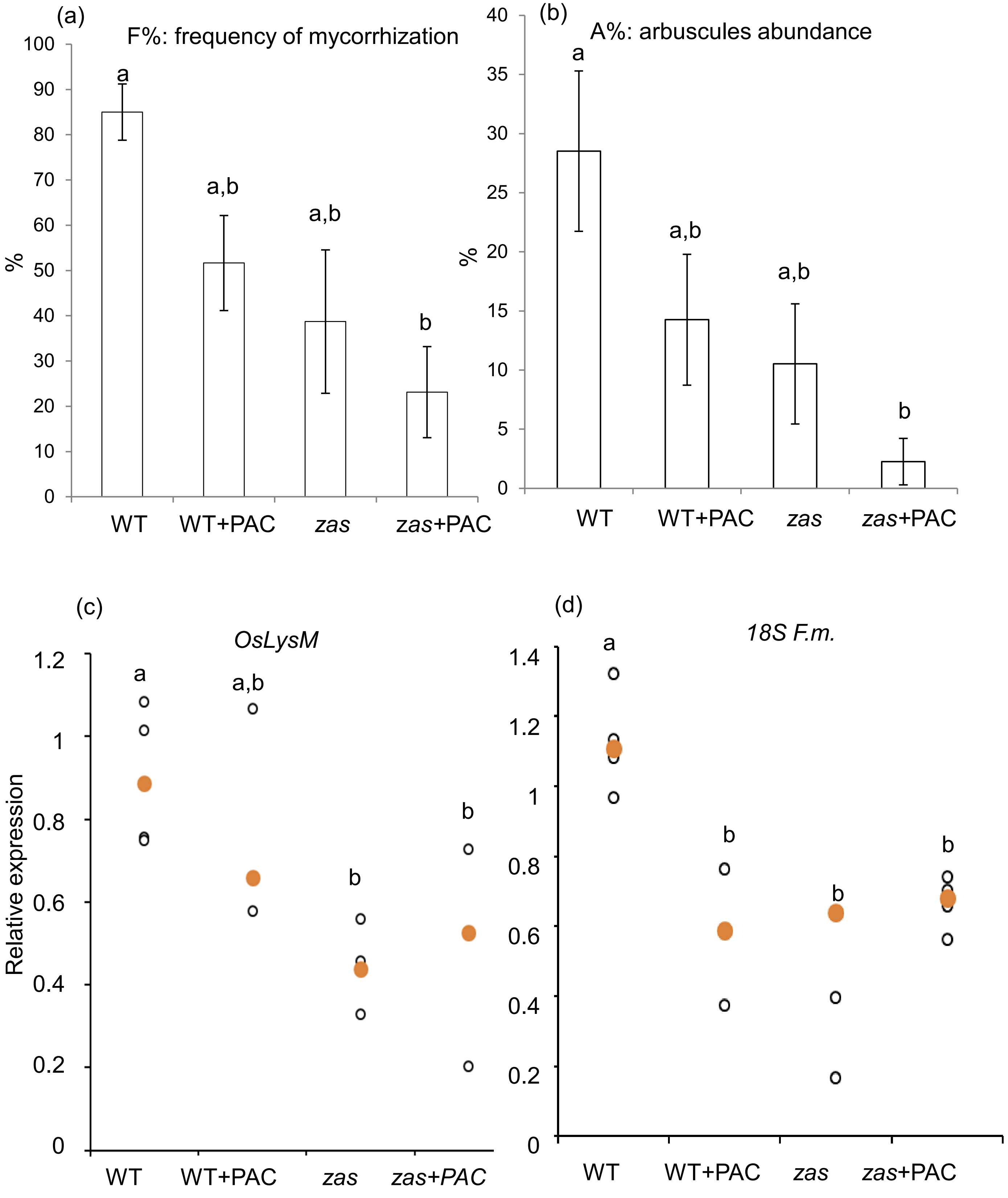
(a)
F%: frequency of mycorrhization
%
a
a,b
a,b
b
WT
WT+PAC
zas
zas+PAC
(b)
A%: arbuscules abundance
%
a
a,b
a,b
b
WT
WT+PAC
zas
zas+PAC
(c)
OsLysM
Relative expression
a
a,b
b
b
WT
WT+PAC
zas
zas+PAC
(d)
18S F.m.
a
b
b
b
WT
WT+PAC
zas
zas+PAC

Figure S6

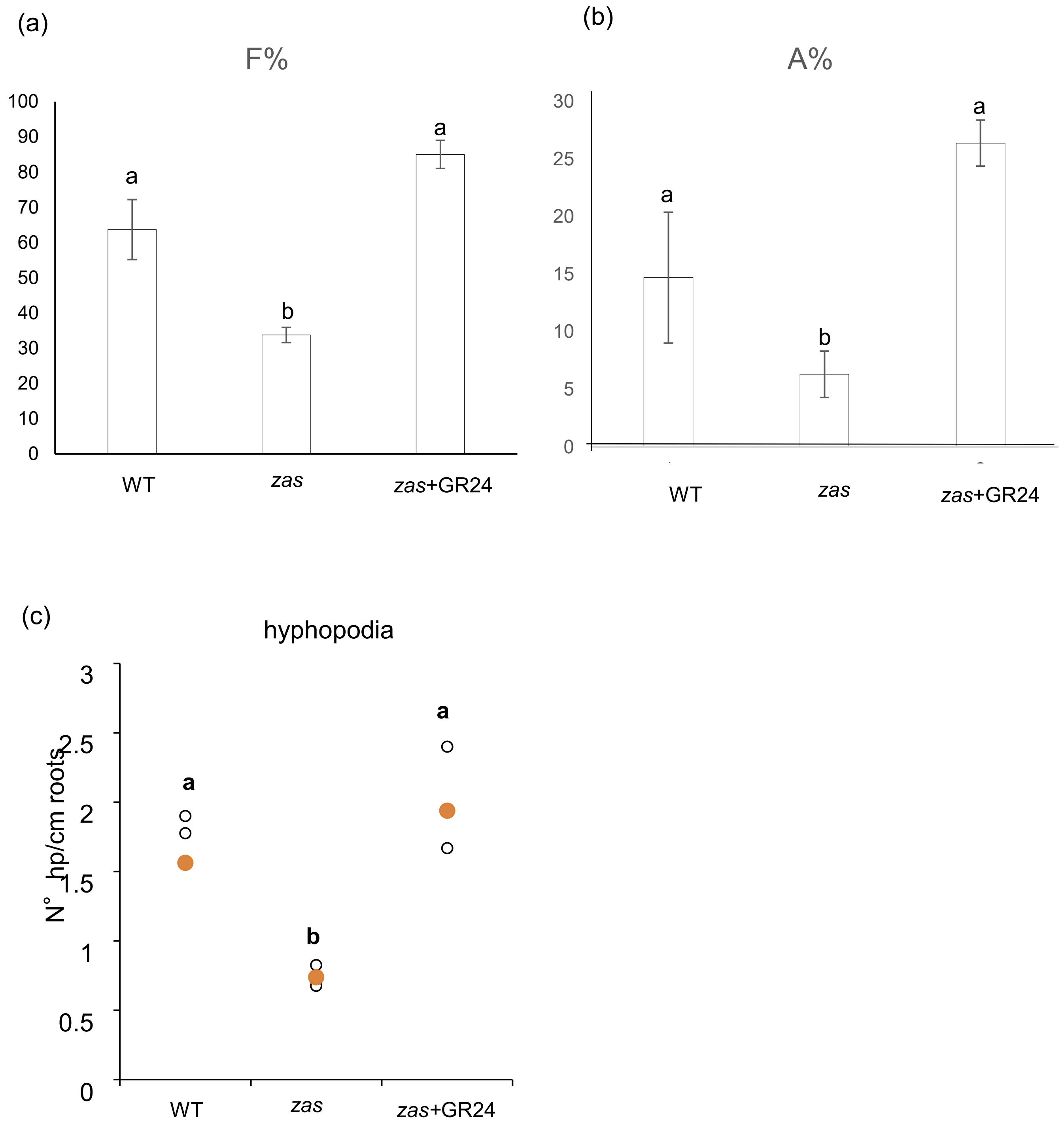
(a)
F%
100
90
80
70
60
50
40
30
20
10
0
a
b
a
WT
zas
zas+GR24
(b)
A%
30
25
20
15
10
5
0
a
b
a
WT
zas
zas+GR24
(c)
hyphopodia
N° hp/cm roots
3
2.5
2
1.5
1
0.5
0
a
b
a
WT
zas
zas+GR24

Figure S7

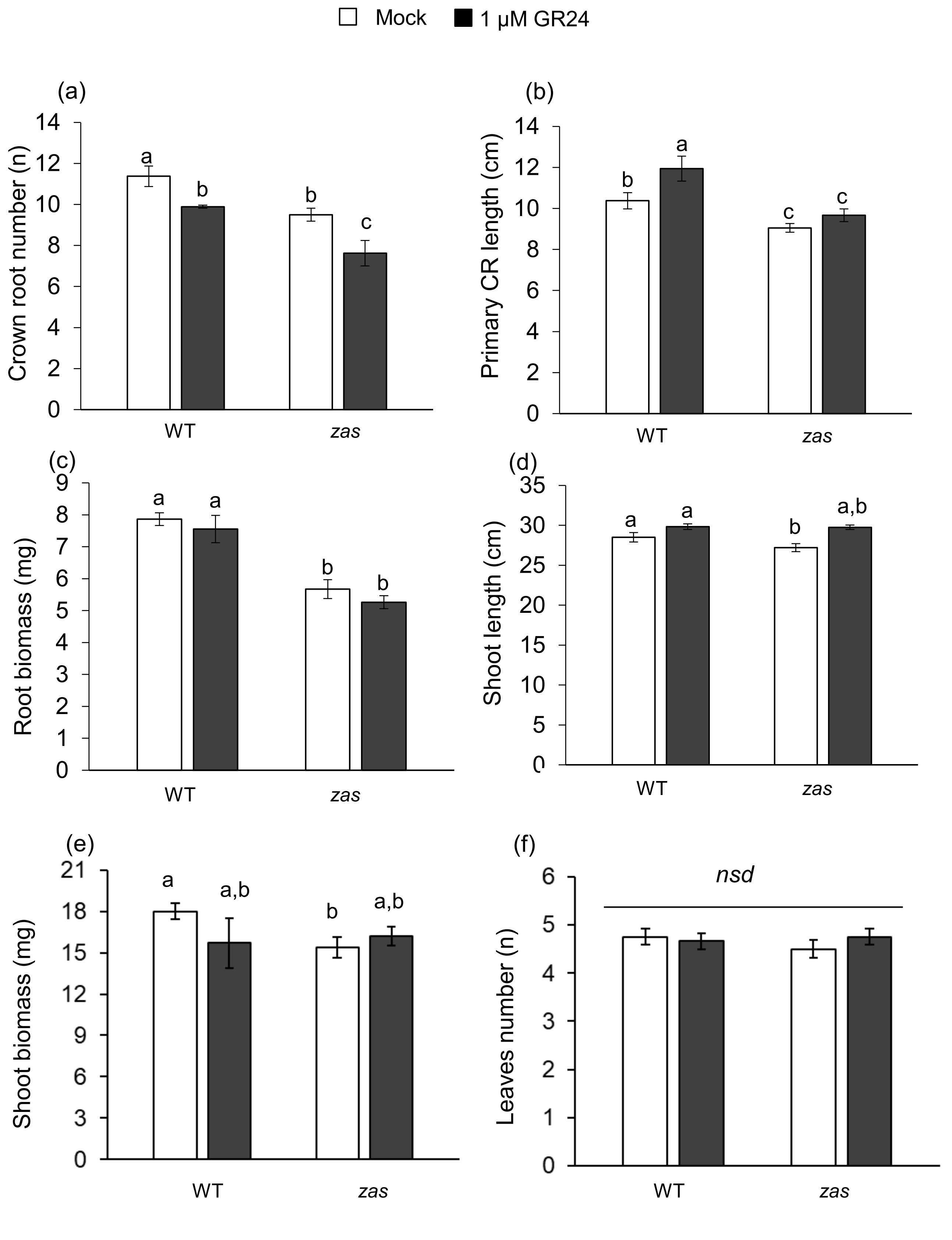
Mock
1 µM GR24
(a)
Crown root number (n)
a
b
b
c
WT
zas
(b)
Primary CR length (cm)
b
a
c
c
WT
zas
(c)
Root biomass (mg)
a
a
b
b
WT
zas
(d)
Shoot length (cm)
a
a
b
a,b
WT
zas
(e)
Shoot biomass (mg)
a
a,b
b
a,b
WT
zas
(f)
Leaves number (n)
nsd
WT
zas

Figure S8

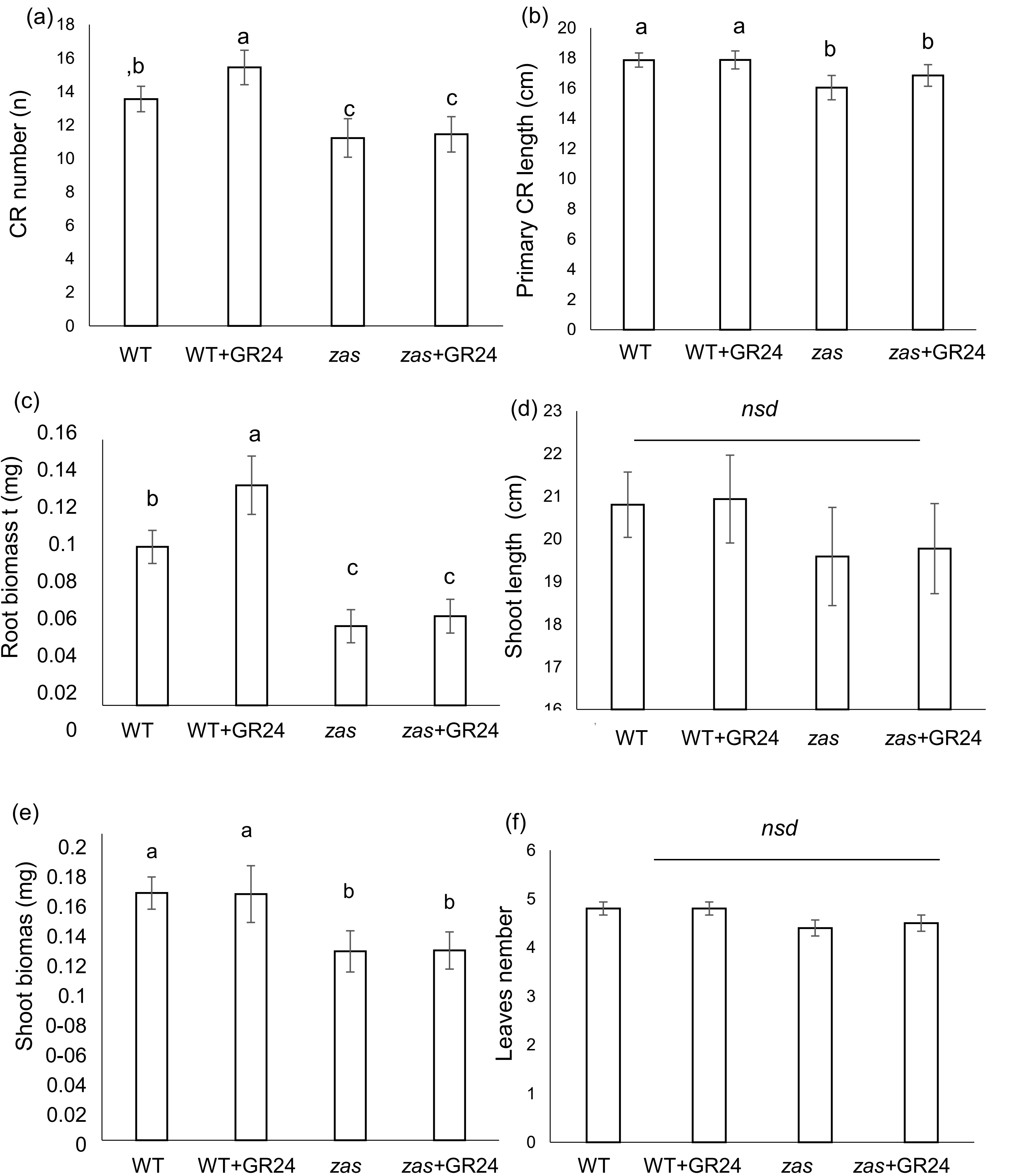
(a)
CR number (n)
18
16
14
12
10
8
6
4
2
0
,b
a
c
c
WT
WT+GR24
zas
zas+GR24
(b)
Primary CR length (cm)
20
18
16
14
12
10
8
6
4
2
0
a
a
b
b
WT
WT+GR24
zas
zas+GR24
(c)
Root biomass t (mg)
0.16
0.14
0.12
0.1
0.08
0.06
0.04
0.02
0
b
a
c
c
WT
WT+GR24
zas
zas+GR24
(d)
nsd
Shoot length (cm)
23
22
21
20
19
18
17
16
WT
WT+GR24
zas
zas+GR24
(e)
Shoot biomas (mg)
0.2
0.18
0.16
0.14
0.12
0.1
0-08
0-06
0.04
0.02
0
a
a
b
b
WT
WT+GR24
zas
zas+GR24
(f)
nsd
Leaves nember
6
5
4
3
2
1
0
WT
WT+GR24
zas
zas+GR24

Figure S9

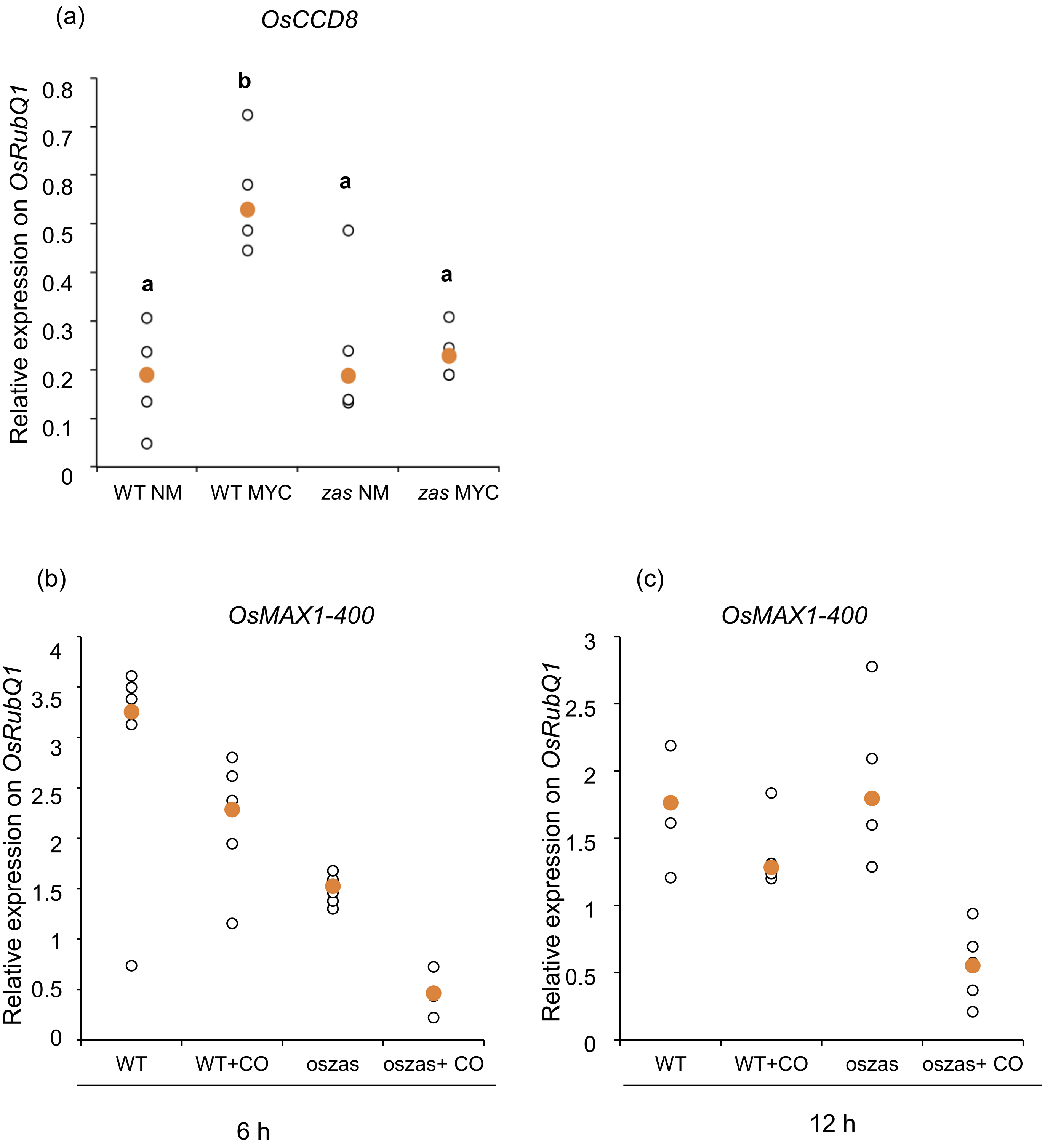
(a)
OsCCD8
Relative expression on OsRubQ1
0.8
0.7
0.8
0.5
0.4
0.3
0.2
0.1
0
b
a
a
a
WT NM
WT MYC
zas NM
zas MYC
(b)
OsMAX1-400
Relative expression on OsRubQ1
4
3.5
3
2.5
2
1.5
1
0.5
0
WT
WT+CO
oszas
oszas+ CO
6 h
(c)
OsMAX1-400
Relative expression on OsRubQ1
3
2.5
2
1.5
1
0.5
0
WT
WT+CO
oszas
oszas+ CO
12 h

Figure S10

(a)

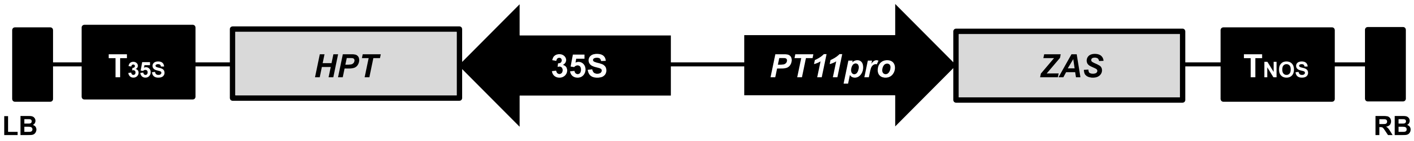


(b)

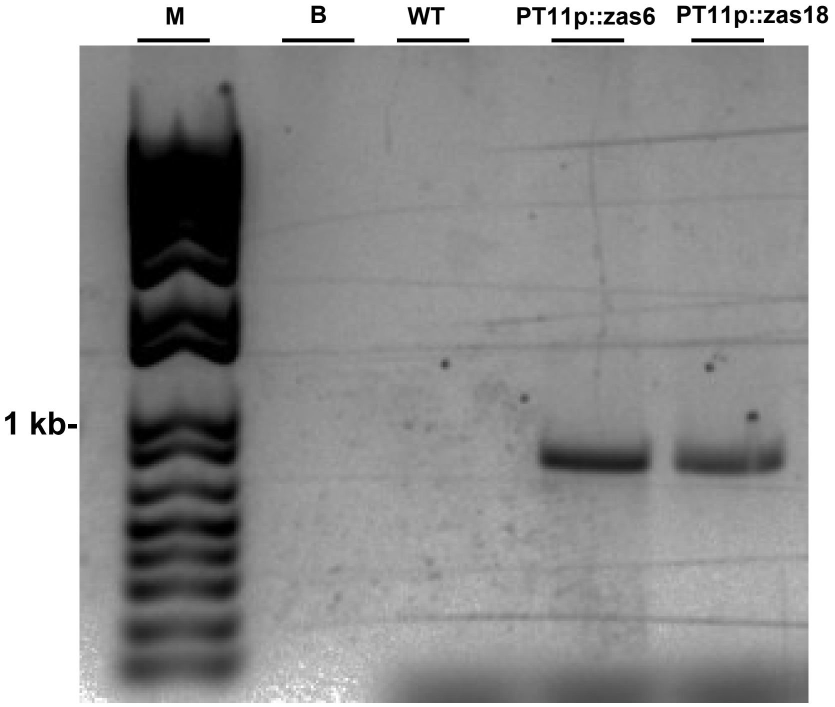


Figure S11

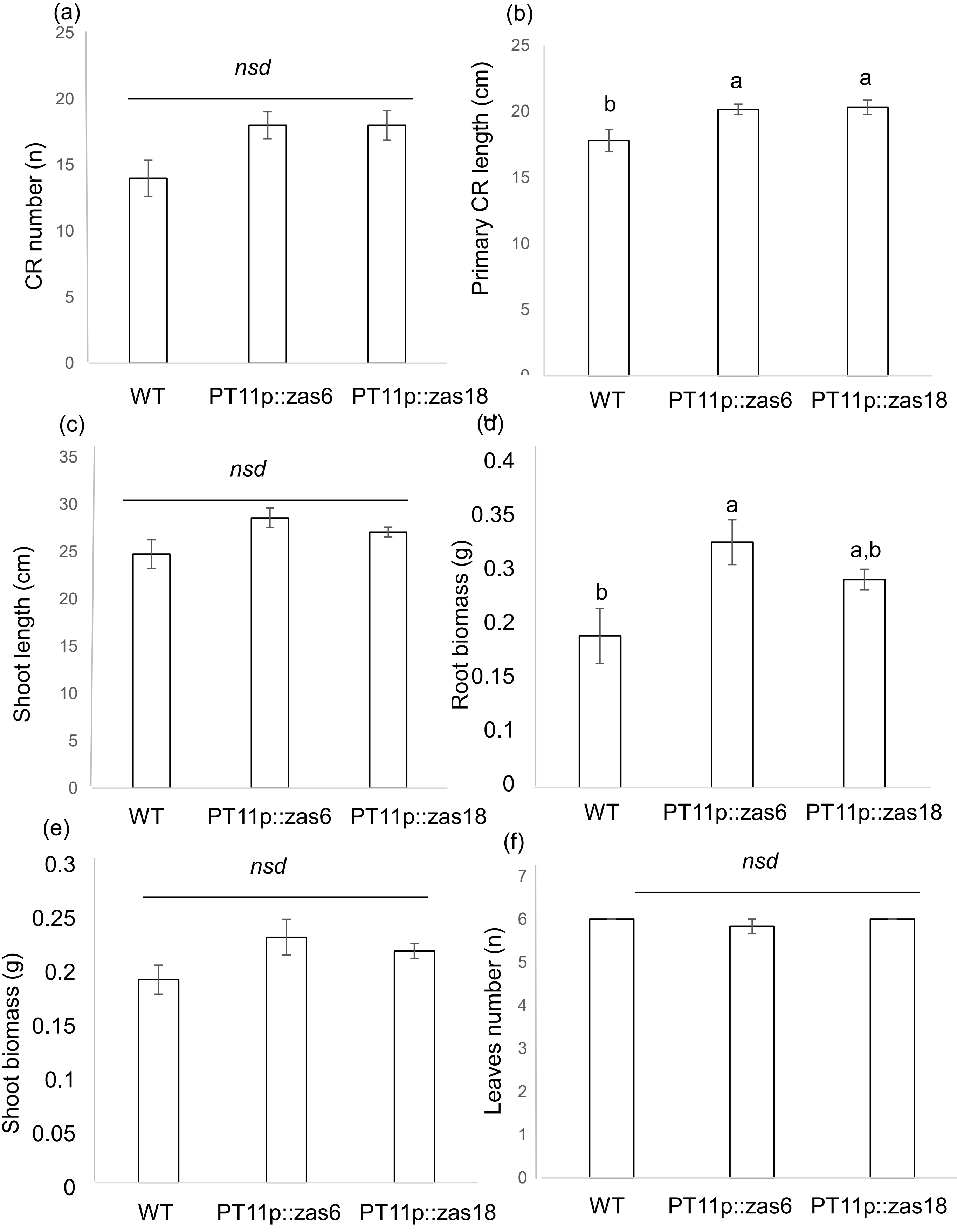
(a)
nsd
CR number (n)
25
20
15
10
5
0
WT
PT11p::zas6
PT11p::zas18
(b)
Primary CR length (cm)
25
20
15
10
5
b
a
a
WT
PT11p::zas6
PT11p::zas18
(c)
nsd
Shoot length (cm)
35
30
25
20
15
10
5
0
WT
PT11p::zas6
PT11p::zas18
(d)
Root biomass (g)
0.4
0.35
0.3
0.2
0.15
0.1
0
b
a
a,b
WT
PT11p::zas6
PT11p::zas18
(e)
nsd
Shoot biomass (g)
0.3
0.25
0.2
0.15
0.1
0.05
0
WT
PT11p::zas6
PT11p::zas18
(f)
nsd
Leaves number (n)
7
6
5
4
3
2
1
0
WT
PT11p::zas6
PT11p::zas18

Figure S12

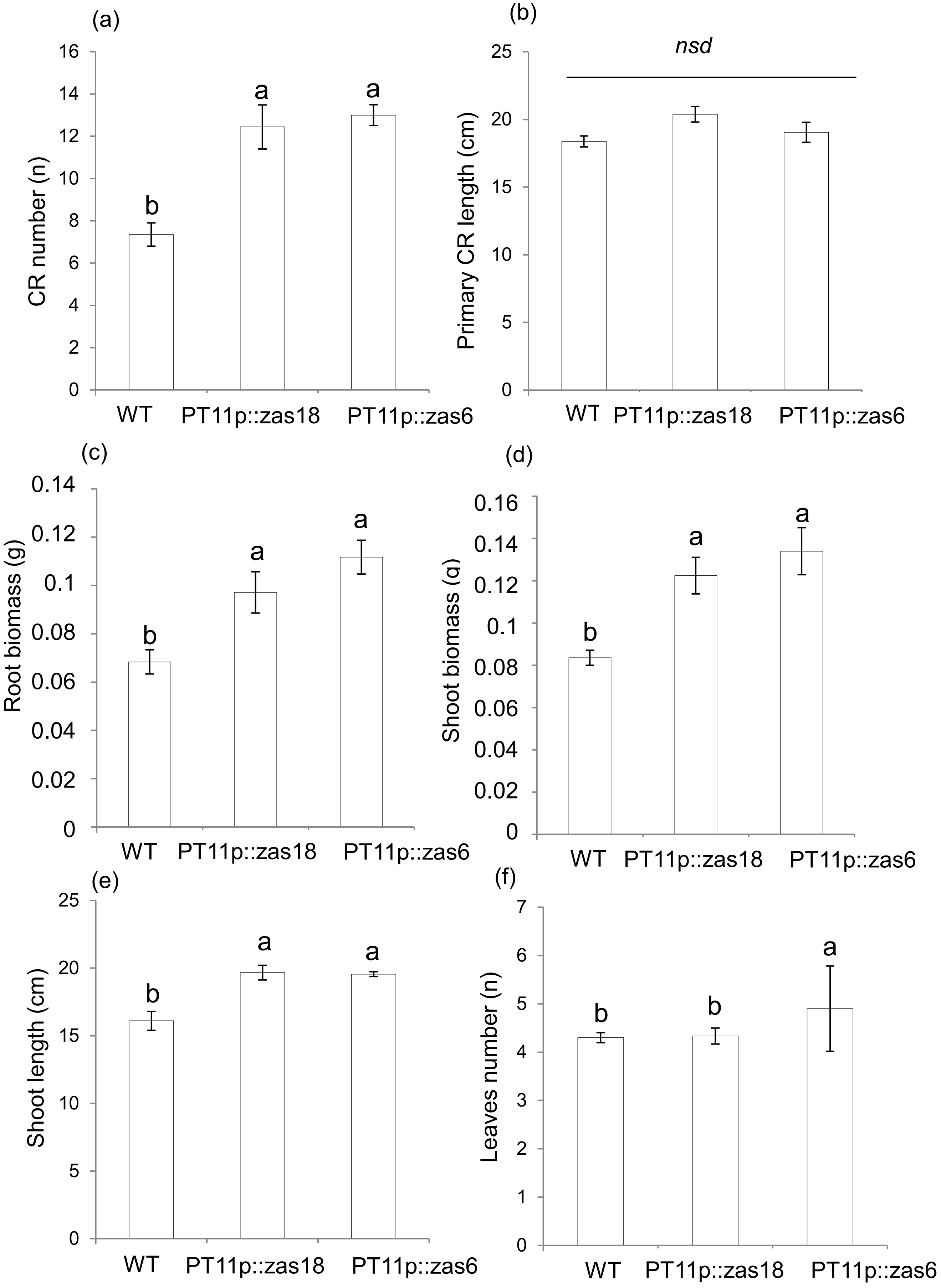

(a)
CR number (n)
0
2
4
6
8
10
12
14
16
b
a
a
WT
PT11p::zas18
PT11p::zas6
(b)
Primary CR length (cm)
nsd
0
5
10
15
20
25
WT
PT11p::zas18
PT11p::zas6
(c)
Root biomass (g)
0
0.02
0.04
0.06
0.08
0.1
0.12
0.14
b
a
a
WT
PT11p::zas18
PT11p::zas6
(d)
Shoot biomass (g)
0
0.02
0.04
0.06
0.08
0.1
0.12
0.14
0.16
b
a
a
WT
PT11p::zas18
PT11p::zas6
(e)
Shoot length (cm)
0
5
10
15
20
25
b
a
a
WT
PT11p::zas18
PT11p::zas6
(f)
Leaves number (n)
0
1
2
3
4
5
6
7
b
b
a
WT
PT11p::zas18
PT11p::zas6


Figure S13

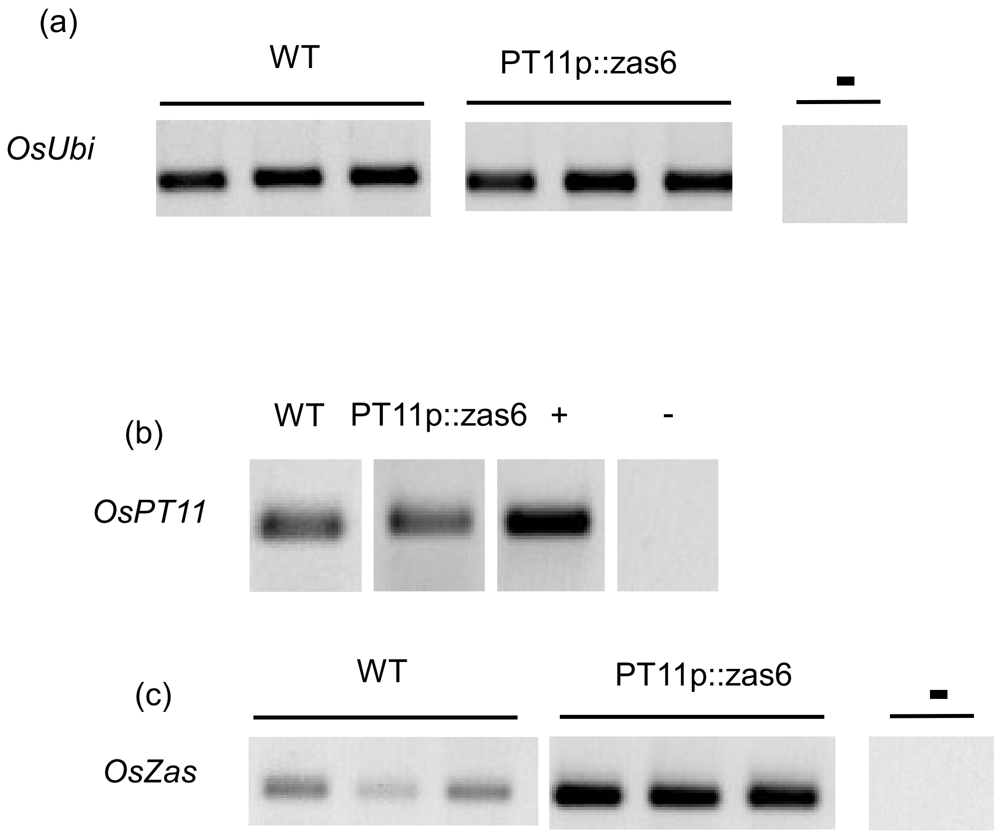
(a)
WT
PT11p::zas6
-
OsUbi
(b)
WT
PT11p::zas6
+
-
OsPT11
(c)
WT
PT11p::zas6
-
OsZas

Figure S14

**Table S1: Phythormones quantification (pmol/g FW) in WT and *zas* non-mycorrhizal and mycorrhizal roots in a time course experiment.**

| | | | | *ABA* | | |
|---|---|---|---|---|---|---|
| | | | ***Sample n*** | ***Raw data*** | ***Mean*** | ***SD*** |
| Roots of Non-mycorrhizal plants | 10DPG | WT | 1 | 16,85 | **18,41 ± 1,01** | |
| | | | 2 | 19,67 | *RSD 5%* | |
| | | | 3 | 18,56 | | |
| | | | 4 | 18,57 | | |
| | | *zas* | 1 | 8,18 | **8,34 ± 0,70** | |
| | | | 2 | 7,82 | *RSD 8%* | |
| | | | 3 | 9,52 | *** / 0,0000 | |
| | | | 4 | 7,83 | | |
| | 20DPG | WT | 1 | 2,86 | **3,36 ± 0,61** | |
| | | | 2 | 4,07 | *RSD 18%* | |
| | | | 3 | 2,67 | | |
| | | | 4 | 3,86 | | |
| | | *zas* | 1 | 6,00 | **5,92 ± 0,48** | |
| | | | 2 | 6,67 | *RSD 8%* | |
| | | | 3 | 5,47 | ** / *0,0015* | |
| | | | 4 | 5,53 | | |
| | 45DPG | WT | 1 | 5,95 | **7,72 ± 1,67** | |
| | | | 2 | 6,49 | *RSD 22%* | |
| | | | 3 | 10,22 | | |
| | | | 4 | 8,22 | | |
| | | *zas* | 1 | 3,35 | **3,31 ± 0,42** | |
| | | | 2 | 3,96 | *RSD 13%* | |
| | | | 3 | 3,14 | * / *0,0165* | |
| | | | 4 | 2,80 | | |
| Roots of mycorrizal plants | 45DPG | WT | 1 | 3,75 | **3,61 ± 0,36** | |
| | | | 2 | 4,07 | *RSD 10%* | |
| | | | 3 | 3,08 | | |
| | | | 4 | 3,55 | | |
| | | *zas* | 1 | 5,41 | **5,29 ± 0,54** | |
| | | | 2 | 6,04 | *RSD 10%* | |
| | | | 3 | 4,53 | ** / *0,0057* | |
| | | | 4 | 5,19 | | |

**Table S2: Gibberellin quantification (pmol/g FW) in WT and *zas* non-mycorrhizal and mycorrhizal roots.**

| | | | | 13-non-hydroxylated gibberellin content (pmol/g DW) | | | | | | | | | | | | | | | | | | | | | |
|---|---|---|---|---|---|---|---|---|---|---|---|---|---|---|---|---|---|---|---|---|---|---|---|---|---|
| | | | ***Sample n*** | $GA_{15}$ | | $GA_{24}$ | | $GA_{13}$ | | $GA_9$ | | $GA_7$ | | $GA_4$ | | $GA_{51}$ | | $GA_{34}$ | | $GA_{20}$ | | $GA_1$ | | $GA_3$ | |
| Roots of non-mycorrhizal plants | 10DPG | WT | 1 | 0,23 | **0,19** | 0,22 | **0,26** | 0,03 | **0,03** | 0,24 | **0,20** | 0,11 | **0,09** | 3,70 | **2,08** | 15,87 | **11,70** | 0,14 | **0,15** | 0,12 | **0,06** | 0,03 | **0,06** | 0,28 | **0,29** |
| | | | 2 | 0,17 | *0,02* | 0,50 | *0,14* | 0,03 | *0,01* | 0,18 | *0,03* | 0,09 | *0,01* | 1,84 | *1,01* | 14,22 | *4,63* | 0,33 | *0,11* | 0,04 | *0,03* | 0,12 | *0,04* | 0,29 | *0,01* |
| | | | 3 | 0,19 | | 0,18 | | 0,04 | | 0,20 | | 0,08 | | 0,92 | | 12,83 | | 0,03 | | 0,05 | | 0,04 | | 0,28 | |
| | | | 4 | 0,17 | | 0,16 | | 0,02 | | 0,18 | | 0,08 | | 1,88 | | 3,90 | | 0,11 | | 0,04 | | 0,06 | | 0,31 | |
| | | *zas* | 1 | 0,36 | **0,21** | 0,23 | **0,17** | 0,02 | **0,03** | 0,41 | **0,28** | 0,13 | **0,10** | 0,97 | **1,77** | 1,04 | **16,51** | 0,27 | **0,17** | 0,21 | **0,21** | 0,07 | **0,06** | 0,30 | **0,32** |
| | | | 2 | 0,14 | *0,09* | 0,13 | *0,04* | 0,06 | *0,02* | 0,15 | *0,13* | 0,09 | *0,03* | 1,77 | *1,13* | 28,54 | *11,65* | 0,11 | *0,06* | 0,22 | *0,01* | 0,10 | *0,03* | 0,34 | *0,02* |
| | | | 3 | 0,16 | | 0,15 | | 0,03 | | *ND* | | 0,11 | | 3,60 | | 9,47 | | 0,12 | | 0,19 | | 0,06 | | 0,31 | |
| | | | 4 | 0,17 | - | 0,16 | - | 0,02 | - | *ND* | - | 0,06 | - | 0,72 | - | 26,97 | - | 0,18 | - | 0,21 | *** | 0,02 | | 0,33 | |
| | 20DPG | WT | 1 | 0,10 | **0,12** | 0,30 | **0,21** | 0,07 | **0,03** | *ND* | **0,46** | 0,06 | **0,06** | 1,17 | **1,95** | 2,94 | **17,00** | 0,03 | **0,07** | 0,20 | **0,20** | 0,01 | **0,03** | 0,21 | **0,21** |
| | | | 2 | 0,13 | *0,01* | 0,13 | *0,09* | 0,02 | *0,02* | 0,30 | *0,16* | 0,09 | *0,01* | 3,07 | *0,69* | 8,89 | *11,28* | 0,03 | *0,05* | 0,21 | *0,03* | 0,05 | *0,02* | 0,20 | *0,01* |
| | | | 3 | 0,11 | | 0,31 | | 0,02 | | 0,62 | | 0,05 | | 1,80 | | 28,31 | | 0,16 | | 0,25 | | 0,01 | | 0,23 | |
| | | | 4 | 0,12 | | 0,11 | | 0,02 | | *ND* | | 0,06 | | 1,76 | | 27,86 | | 0,08 | | 0,15 | | 0,03 | | 0,20 | |
| | | *zas* | 1 | 0,74 | **0,46** | 0,49 | **0,42** | 0,87 | **0,40** | 0,13 | **0,21** | 0,75 | **0,78** | 12,47 | **11,03** | 7,96 | **6,68** | 0,17 | **0,21** | 1,06 | **1,08** | 0,05 | **0,14** | 1,11 | **1,10** |
| | | | 2 | 0,20 | *0,29* | 0,59 | *0,16* | 0,20 | *0,29* | 0,21 | *0,08* | 0,71 | *0,37* | 9,51 | *2,65* | 12,52 | *3,96* | 0,28 | *0,04* | 1,20 | *0,08* | 0,19 | *0,07* | 1,10 | *0,29* |
| | | | 3 | 0,77 | | 0,15 | | 0,12 | | 0,35 | | 0,31 | | 7,62 | | 2,18 | | 0,19 | | 1,11 | | 0,10 | | 0,69 | |
| | | | 4 | 0,15 | * | 0,44 | - | 0,39 | * | 0,16 | - | 1,35 | * | 14,51 | *** | 4,08 | - | 0,22 | ** | 0,97 | *** | 0,22 | * | 1,52 | *** |
| | 45DPG | WT | 1 | 0,13 | **0,41** | 3,83 | **3,25** | 0,02 | **0,02** | 0,92 | **0,52** | 0,08 | **0,07** | 2,85 | **2,21** | 8,49 | **9,00** | 0,31 | **0,21** | 0,43 | **0,35** | 0,03 | **0,01** | 0,23 | **0,22** |
| | | | 2 | 0,54 | *0,32* | 2,04 | *0,76* | ~~0,02~~ | *0,00* | 0,65 | *0,30* | 0,04 | *0,02* | 1,95 | *0,38* | 7,80 | *2,05* | 0,12 | *0,12* | 0,22 | *0,08* | 0,01 | *0,01* | 0,23 | *0,01* |
| | | | 3 | 0,08 | | 3,95 | | 0,02 | | 0,35 | | 0,07 | | 1,90 | | 12,47 | | 0,07 | | 0,38 | | 0,01 | | 0,21 | |
| | | | 4 | 0,87 | | 3,19 | | 0,02 | | 0,13 | | 0,08 | | 2,14 | | 7,23 | | 0,35 | | 0,37 | | 0,01 | | 0,21 | |
| | | *zas* | 1 | 0,15 | **0,25** | 1,77 | **3,61** | 0,36 | **0,31** | 0,15 | **0,14** | 0,09 | **0,10** | 3,25 | **1,63** | 3,66 | **2,82** | 0,24 | **0,21** | 1,78 | **1,69** | 0,02 | **0,03** | 0,38 | **0,41** |
| | | | 2 | 0,18 | *0,11* | 3,44 | *1,45* | 0,26 | *0,09* | 0,20 | *0,04* | 0,14 | *0,02* | 1,64 | *1,05* | 2,18 | *0,82* | 0,17 | *0,03* | 1,75 | *0,17* | 0,03 | *0,01* | 0,47 | *0,06* |
| | | | 3 | 0,43 | | 5,84 | | 0,42 | | 0,09 | | 0,10 | | 1,28 | | 3,60 | | 0,19 | | 1,84 | | 0,04 | | 0,46 | |
| | | | 4 | 0,24 | - | 3,40 | - | 0,20 | *** | 0,13 | - | 0,09 | * | 0,33 | - | 1,86 | ** | 0,25 | - | 1,39 | *** | 0,04 | | 0,32 | ** |
| Roots of mycorrizal roots | 45DPG | WT | 1 | 0,29 | **0,39** | 5,77 | **6,50** | 0,02 | **0,02** | 0,32 | **0,39** | 0,06 | **0,06** | 3,10 | **1,11** | 2,84 | **8,85** | 0,25 | **0,21** | 0,04 | **0,07** | 0,01 | **0,02** | 0,19 | **0,21** |
| | | | 2 | 0,56 | *0,15* | 5,38 | *1,58* | 0,02 | *0,00* | 0,15 | *0,18* | 0,08 | *0,01* | 0,31 | *1,15* | 11,61 | *6,08* | 0,18 | *0,05* | 0,07 | *0,02* | 0,04 | *0,01* | 0,23 | *0,01* |
| | | | 3 | 0,20 | | 9,23 | | 0,02 | | 0,45 | | 0,07 | | 0,58 | | 17,51 | | 0,27 | | 0,06 | | 0,01 | | 0,21 | |
| | | | 4 | 0,52 | | 5,62 | | 0,02 | | 0,63 | | 0,05 | | 0,44 | | 3,43 | | 0,14 | | 0,10 | | 0,01 | | 0,20 | |
| | | *zas* | 1 | 0,16 | **0,16** | 1,13 | **1,75** | 0,02 | **0,03** | 0,98 | **0,84** | 0,06 | **0,06** | 0,35 | **1,14** | 10,06 | **7,82** | 0,22 | **0,20** | 0,18 | **0,17** | 0,03 | **0,06** | 0,28 | **0,22** |
| | | | 2 | 0,16 | *0,01* | 1,13 | *0,62* | 0,03 | *0,00* | 0,98 | *0,26* | 0,04 | *0,01* | 0,36 | *0,83* | 7,63 | *2,39* | 0,17 | *0,08* | 0,14 | *0,06* | 0,02 | *0,04* | 0,21 | *0,04* |
| | | | 3 | 0,14 | | 2,40 | | 0,02 | | 1,01 | | 0,07 | | 1,55 | | 4,00 | | 0,10 | | 0,26 | | 0,13 | | 0,21 | |
| | | | 4 | 0,18 | * | 2,33 | ** | 0,02 | - | 0,40 | * | 0,06 | - | 2,29 | - | 9,58 | - | 0,32 | - | 0,10 | * | 0,08 | * | 0,17 | |

**Table S3. Primer sequences used in this study**

| Experiment | Primer name | Sequence (5′–3′) | Restriction enzyme |
|---|---|---|---|
| For *pCAMB1300-OsPT11prom:OsZAS* construct | *ZAS-F* | ATGATGACAGCTTCTCTGCA | |
| | *ZAS-R* | TCACGTCATTGTGTTTGCTAG | |
| | *Pro-PT11-F* | ACT<u>GAATTC</u>GTACAAATTCTTCATTTTTTTAGCGAAGTTAGAAG | <u>*EcoRI*</u> |
| | *Pro-PT11-R* | CGT<u>TCTAGA</u>CTCCGATGATGCCGTCGAT | <u>*XbaI*</u> |
| For *pCAMB1300-OsPT11prom:OsZAS* transgenicity PCR test | *PT11prom-Fwd* | CTTGCAAGGGACAAAACAC | |
| | *ZAS-Rev* | AGTGGATTGGAACCATTTCT | |
| For gene expression analysis (RT-qPCR) | *OsZAS* | F:CTCCATTCGACCGTCTCAATCT | |
| | | R:TGGCTTCTGTCTGGTTTTCTCA | |
| | *OsLysM* | F:CGCTGACATGCAACAAGGTG | |
| | | R:CTTCGCGCAGTTGATGTTTGG | |
| | *OsPT11* | F: GAGAAGTTCCCTGCTTCAAGCA | |
| | | R: GAGAAGTTCCCTGCTTCAAGCA | |
| | *OsRubQ1* | F:GGGTTCACAAGTCTGCCTATTTG | |
| | | R: ACGGGACACGACCAAGGA | |
| | *Fm. 18S* | F: GCTATTTTGATCATTGCCGCC | |
| | | R: TCATTAAAACGTTCTTCCGACC | |
| | *Osccd8* | F: GTTCCAGTACACGGACAAG | |
| | | R: ACTGCCTCTCGTTGCTA | |
| | *OsMAX1-1400* | F: ACGACGGCGTTCACTCTCTC | |
| | | R: TCCGAACCCGTCAATCTCC | |
| | *OsD14L* | F: GAAGCCATGGGGTCAAACTA | |
| | | R: GCGGCTAAACTCCTGAACTG | |
| | *OsSMAX1* | F: AGGCATGGAGGTTATAGATCT | |
| | | R: ACCGATCAATCCTTGCAACA | |
| For probe generation for the *in situ* hybridization | *M13-F* | GTAAAACGACGGCCAGT | |
| | *OsZAS-SP6-R* | CTATTTAGGTGACACTATAGAcaccctcttcaaagcagttgat | |
