# Supplementary material for: Zaxinone synthase controls arbuscular mycorrhizal colonization level in rice
Source: Plant J. 2022 Aug 17;111(6):1688–700. doi: 10.1111/tpj.15917 (PMC9543690; doi:10.1111/tpj.15917)
Supplement: Supplementary file 2 — Supplemental methods [file TPJ-111-1688-s002.docx]

**Supplemental methods**

*OsPT11prom::*Oszas *lines generation*

The construct in which ZAS was expressed under the mycorrhizal-specific promoter OsPT11 was made in the binary vector pCAMBIA1300 (see Supplemental Methods) by cloning two different fragments as following: the full-length cDNA of ZAS (LOC_Os09g15240) was PCR-amplified using primers ZAS-F and ZAS-R (Table S3); a 1360 bp promoter fragment upstream of the translation start codon of OsPT11 (LOC_Os01g46860) was PCR-amplified from Nipponbare genomic DNA using primers Pro-PT11-F and Pro-PT11-R (Table S3). After confirmation by sequencing, finally an *Eco*RI/*Xba*I OsPT11-promoter fragment was cloned into binary vector pCAMB1300-ZAS cut with *Eco*RI/*Xba*I, in which 35S promoter was replaced with OsPT11 promoter, resulting in pCAMB1300-PT11:ZAS plasmid. The plasmid was introduced into Nipponbare wild type Japonica rice cultivar by *Agrobacterium tumefaciens* (strain EHA105)-mediated transformation as previously described (Hiei et al. 2008).

In Situ *hybridization*

*Plant material Sample Fixation and Embedding*

Seeds of WT cv Nipponbare were germinated in pots containing sterile quartz sand and incubated for 7 d in a growth chamber under a 14 h light (23 °C)/10 h dark (21 °C). Plants were inoculated with a Funneliformis mosseae fungal inoculum (BEG 12, MycAgroLab, France) mixed (25%) with sand. Plants were grown and watered twice a week: once with tap water and once with a modified Long-Ashton solution (32 µM Na_2_HPO4·12H_2_O; Hewitt 1966). Non – mycorrhizal and mycorrhizal roots were harvested at 35 dpi (days post inoculation).

 Inoculated and non-inoculated roots were fixed in 4% paraformaldehyde in PBS (phosphate buffered saline: 130 mM NaCl; 7 mM Na_2_HPO_4_; 3 mM NaH_2_PO_4_, pH 7.4) overnight at 4°C. For the first 15 to 30 min, samples were fixed under vacuum to facilitate infiltration with the fixative. The tissue was then dehydrated in successive steps, each of 30 to 60 min duration, in 30%, 50%, 70%, 80%, 95%, and 100% ethanol and 100% xylene. Finally, the samples were embedded in paraffin wax (Paraplast plus; Sigma) at 60°C. Sections of 7 to 8 mm were then transferred to slides treated with 100 mg/mL poly-L-Lys (Sigma) and dried on a warm plate at 40°C overnight.

*Preparation of probes*

DIG-labeled RNA probes were synthesized starting with 1 mg of PCR-obtained template (Langdale, 1993). DIG-labeled riboprobes (antisense and sense probes) were produced with DIG-UTP by *in vitro* transcription using the Sp6 and T7 promoters according to the manufacturer’s protocol (RNA-labeling kit; Roche).

*In Situ* Hybridization and Detection

The sections were treated as follows: deparaffinized in Neoclear, rehydrated through an ethanol series, treated with 0.2 M HCl for 20 min, washed in sterile water for 5 min, incubated in 23 SSC for 10 min, washed in sterile water for 5 min, incubated with proteinase K (1 mg/mL in 100 mM Tris-HCl, pH 8.0, 50 mM EDTA; Roche) at 37°C for 30 min, washed briefly in PBS, and then treated with 0.2% glycine in PBS for 5 min. After two rinses in PBS, slides were incubated in 4% paraformaldehyde in PBS for 20 min, washed in PBS (2 3 5 min), and then dehydrated in an ethanol series from 30% to 100%. Hybridizations were carried out overnight at 55°C with denatured DIG-labeled RNA probes in 50% formamide, 20X SSC, 20% SDS, 50 mg/mL tRNA, 40 μg/ml Salmon Sperm DNA. Slides were then washed twice in 13 SSC, 0.1% SDS at room temperature and rinsed with 0.23 SSC, 0.1% SDS at 55 C (2 3 10 min). After rinsing with 23 SSC for 5 min at room temperature, the non-specifically bound DIG-labeled probe was removed by incubating in 10 mg/mL RNase A in 23 SSC at 37°C for 30 min. Slides were then rinsed twice in 2% SSC before proceeding to the next stage. The hybridized probe was detected using an alkaline phosphatase antibody conjugate (Roche). After rinsing in TBS (100 mM Tris-HCl, pH 7.5, 400 mM NaCl) for 5 min, slides were treated with 0.5% blocking reagent in TBS for 1 h, incubated for 2 h with the anti-DIG alkaline phosphatase conjugate diluted 1:500 in 0.5% BSA Fraction V in TBS, and then washed in TBS (3 x 5 min). Color development was carried out according to Torres et al. (1995). The color reaction was stopped by washing in distilled water, and the sections were then dehydrated through an ethanol series, deparaffinized in Neoclear, and mounted in Neomount (Merck).

**References**

**Hiei Y, Komari T. 2008.** Agrobacterium-mediated transformation of rice using immature embryos or calli induced from mature seed. *Nature Protocols* **3**: 824–834.
